# Objective assessment of dietary patterns by use of metabolic phenotyping: a randomised, controlled, crossover trial

**DOI:** 10.1016/S2213-8587(16)30419-3

**Published:** 2017-03

**Authors:** Isabel Garcia-Perez, Joram M Posma, Rachel Gibson, Edward S Chambers, Tue H Hansen, Henrik Vestergaard, Torben Hansen, Manfred Beckmann, Oluf Pedersen, Paul Elliott, Jeremiah Stamler, Jeremy K Nicholson, John Draper, John C Mathers, Elaine Holmes, Gary Frost

**Affiliations:** a Nutrition and Dietetic Research Group, Division of Endocrinology and Metabolism, Department of Medicine, Imperial College London, London, UK; b Biomolecular Medicine, Division of Computational and Systems Medicine, Department of Surgery and Cancer, Imperial College London, London, UK; c Department of Epidemiology and Biostatistics, Medical Research Council (MRC)–Public Health England Centre for Environment and Health, School of Public Health, Imperial College London, London, UK; d MRC–National Institute for Health Research (NIHR) National Phenome Centre, Department of Surgery and Cancer, Imperial College London, London, UK; e The Novo Nordisk Foundation Center for Basic Metabolic Research, Section of Metabolic Genetics, Faculty of Health Sciences, University of Copenhagen, Copenhagen, Denmark; f Faculty of Health Sciences, University of Southern Denmark, Odense, Denmark; g Institute of Biological, Environmental and Rural Sciences, Aberystwyth University, Aberystwyth, UK; h Department of Preventive Medicine, Feinberg School of Medicine, Northwestern University, Chicago, USA; i Human Nutrition Research Centre, Institute of Cellular Medicine, Newcastle University, Newcastle, UK

## Abstract

**Background:**

Accurate monitoring of changes in dietary patterns in response to food policy implementation is challenging. Metabolic profiling allows simultaneous measurement of hundreds of metabolites in urine, the concentrations of which can be affected by food intake. We hypothesised that metabolic profiles of urine samples developed under controlled feeding conditions reflect dietary intake and can be used to model and classify dietary patterns of free-living populations.

**Methods:**

In this randomised, controlled, crossover trial, we recruited healthy volunteers (aged 21–65 years, BMI 20–35 kg/m^2^) from a database of a clinical research unit in the UK. We developed four dietary interventions with a stepwise variance in concordance with the WHO healthy eating guidelines that aim to prevent non-communicable diseases (increase fruits, vegetables, whole grains, and dietary fibre; decrease fats, sugars, and salt). Participants attended four inpatient stays (72 h each, separated by at least 5 days), during which they were given one dietary intervention. The order of diets was randomly assigned across study visits. Randomisation was done by an independent investigator, with the use of opaque, sealed, sequentially numbered envelopes that each contained one of the four dietary interventions in a random order. Participants and investigators were not masked from the dietary intervention, but investigators analysing the data were masked from the randomisation order. During each inpatient period, urine was collected daily over three timed periods: morning (0900–1300 h), afternoon (1300–1800 h), and evening and overnight (1800–0900 h); 24 h urine samples were obtained by pooling these samples. Urine samples were assessed by proton nuclear magnetic resonance (^1^H-NMR) spectroscopy, and diet-discriminatory metabolites were identified. We developed urinary metabolite models for each diet and identified the associated metabolic profiles, and then validated the models using data and samples from the INTERMAP UK cohort (n=225) and a healthy-eating Danish cohort (n=66). This study is registered with ISRCTN, number ISRCTN43087333.

**Findings:**

Between Aug 13, 2013, and May 18, 2014, we contacted 300 people with a letter of invitation. 78 responded, of whom 26 were eligible and invited to attend a health screening. Of 20 eligible participants who were randomised, 19 completed all four 72 h study stays between Oct 2, 2013, and July 29, 2014, and consumed all the food provided. Analysis of ^1^H-NMR spectroscopy data indicated that urinary metabolic profiles of the four diets were distinct. Significant stepwise differences in metabolite concentrations were seen between diets with the lowest and highest metabolic risks. Application of the derived metabolite models to the validation datasets confirmed the association between urinary metabolic and dietary profiles in the INTERMAP UK cohort (p<0·0001) and the Danish cohort (p<0·0001).

**Interpretation:**

Urinary metabolite models developed in a highly controlled environment can classify groups of free-living people into consumers of diets associated with lower or higher non-communicable disease risk on the basis of multivariate metabolite patterns. This approach enables objective monitoring of dietary patterns in population settings and enhances the validity of dietary reporting.

**Funding:**

UK National Institute for Health Research and UK Medical Research Council.

## Introduction

So-called western dietary patterns (ie, high in saturated fat, cholesterol, sodium, and added sugars; low in fruits, vegetables, and fibre) increase the risk of obesity and many non-communicable diseases, including diabetes, coronary heart disease, and cancers.^1^, ^2^, ^3^, ^4^ Overall dietary patterns might be more informative about non-communicable disease risk than individual foods or nutrients.^5^ Many governments have introduced population-based policies aiming to improve dietary patterns and reduce disease burden. These policies have a common core goal (reflected in the WHO Global Strategy on Diet, Physical Activity and Health^6^) of decreasing added sugar, sodium, and total fat consumption, and increasing intakes of wholegrain cereals, fruits, vegetables, and fibre. Results from the North Karelia project^7^ showed that such dietary change can contribute to decreased coronary heart disease mortality at the population level.

**Research in context**

**Evidence before this study**

We searched PubMed for studies published in English from database inception to Oct 6, 2016, using the search terms “metabolomics OR metabonomics OR metabolic profiling OR metabolomic OR metabonomic OR metabolite profiling” and “dietary intervention OR dietary intake OR dietary pattern” and excluding non-human studies and review, perspective, opinion, comment, and protocol articles. The search identified 58 studies, of which 45 were related to associations between dietary patterns and metabolite profiles. 27 of these studies were related to consumption of dietary patterns rather than supplementation of a standard diet with a specific food. All 27 studies used self-reported dietary data obtained with instruments such as food frequency questionnaires, dietary recall, and diet diaries, which can be prone to misreporting, with under-reporting biased towards unhealthy foods and over-reporting towards fruits and vegetables. Such inadequate dietary reporting in epidemiological surveys has led to conflicting research findings.

We did not find any studies that investigated associations between dietary patterns and metabolic profiles in a controlled crossover clinical trial setting. Established dietary biomarkers such as urinary sodium, potassium, and nitrogen track intake of specific nutrients only, and although a few reports have linked specific metabolite profiles to dietary patterns, these studies used self-reported food intake only. Urine and plasma have been shown to contain individual metabolites that are reflective of self-reported food intake data. In this study, we aimed to develop a new approach to assess dietary patterns using proton nuclear magnetic resonance (^1^H-NMR) spectroscopic profiling of urine.

**Added value of this study**

We developed a metabolic profiling strategy, using NMR spectroscopy of urine samples, that can objectively assess dietary profiles. We have shown that urinary metabolite models, developed in a highly controlled environment, can be used to classify free-living people into consumers of a dietary profile associated with low or high risk of non-communicable disease. Previous studies that related dietary patterns to metabolic profiles relied on self-reported dietary data or were performed in a non-controlled setting (eg, a home environment), which increases the possibility of misreporting and non-compliance. Our study was performed in a controlled environment to guarantee compliance and eliminate misreporting, and we validated our model using both internal controlled clinical trial data and external cohort data. This approach has the potential to be used to monitor dietary patterns objectively in population settings without the risk of making false inferences based on data prone to misreporting or non-compliance, which can confound the true effect of a diet on health.

**Implications of all the available evidence**

Unhealthy dietary patterns are major risk factors of multiple common diseases, and many countries have local and national health policies that encourage dietary change. However, existing dietary tools are inadequate for assessing responses in dietary behaviour that result from policy change. Implementation of our metabolic profiling approach and analyses of dietary patterns based on urine metabolite profiles might not only enhance understanding of the relations between diet and health but also offer an objective method for dietary screening of large numbers of people. Our metabolic profiling strategy could be used to obtain objective information on adherence to healthy eating programmes aimed at combating obesity and common diseases.

A major limitation of nutritional science is the objective assessment of dietary intake in free-living populations. Monitoring of dietary change in national surveys and large prospective studies relies on self-reported food intake using instruments such as food frequency questionnaires, dietary recall, and diet diaries; the prevalence of misreporting with these tools is estimated at 30–88%.^8^, ^9^ Compounding this problem, bias in dietary misreporting (with under-reporting biased towards unhealthy foods and over-reporting towards fruits and vegetables^10^) contributes to data inaccuracy and misinterpretation.^11^, ^12^ Moreover, under-reporting of dietary energy intake is particularly common in obese individuals,^8^ which is a major concern considering the increasing prevalence of obesity worldwide.^13^

Established dietary biomarkers such as urinary sodium, potassium, and nitrogen track intake of specific nutrients only. To our knowledge, no independent, objective method exists for assessing overall dietary patterns in free-living populations. This limitation has led to conflicting research findings,^12^ partly because of inadequate dietary reporting in epidemiological surveys.^14^ Urine and plasma have been shown to contain individual metabolites that are reflective of self-reported food intake data.^15^, ^16^, ^17^, ^18^ We now propose a new approach to assess dietary patterns using proton nuclear magnetic resonance (^1^H-NMR) spectroscopic profiling. This technology has potential to simultaneously measure hundreds of metabolites, the concentrations of which are affected by food intake.^19^ If validated, this approach could enhance understanding of the relation between food consumption and disease risk, a concept embedded in the Precision Medicine Initiative.^20^ Although a few reports^15^, ^16^, ^17^, ^18^ have linked metabolite profiles to dietary patterns, these studies used self-reported food intake. To address dietary misreporting by free-living populations,^8^, ^9^ we hypothesised that metabolic profiles of urine from volunteers exposed to a range of diets based on WHO dietary guidelines^6^ for the prevention of non-communicable disease risks (obesity, diabetes, and coronary heart disease), under highly controlled conditions, reflect dietary intake and can be used to model and classify dietary patterns of free-living people from large cross-sectional cohorts, without requiring self-reported food intake. In this study, we aimed to quantify the effect of diverse dietary patterns on urinary metabolic profiles in a highly controlled environment.

## Methods

### Study design and participants

In this randomised, controlled, crossover trial, we recruited participants from a database of healthy volunteers at the UK National Institute for Health Research (NIHR)/Wellcome Trust Imperial Clinical Research Facility (CRF). These volunteers had previously been screened at the CRF and had expressed an interest in being contacted regarding future research studies. Volunteers in the database who were aged 21–65 years and had a BMI of 20–35 kg/m^2^ were contacted with a letter of invitation, and those who responded with an interest in participation were screened, initially by email or telephone and subsequently at the CRF. Potential participants were excluded if they had clinically significant illnesses, if they reported weight loss or gain of 3 kg or more in the preceding 2 months, if they were taking prescription medication, if they were current smokers or substance abusers, or if they presented any abnormalities on physical examination, electrocardiography, or screening blood tests. Women were ineligible if they were pregnant or breastfeeding.

The study was approved by the London–Brent Research Ethics Committee and done in accordance with the Declaration of Helsinki (13/LO/0078). The study protocol is available in the appendix. All participants provided written informed consent.

### Randomisation and masking

Participants were given one dietary intervention during each inpatient stay. The order of diets was randomly assigned across study visits. Randomisation was done by an investigator who was not directly involved in the study, with the use of opaque, sealed, sequentially numbered envelopes that each contained one of the four dietary interventions in a random order. The envelopes were stored securely, away from the trial site, and opened in sequence by an investigator (ESC) as each participant was enrolled. Although participants and investigators could not be masked from the dietary intervention during the study period, investigators analysing the data were masked from the randomisation order.

### Procedures

Participants attended the CRF for a 72 h inpatient period on four occasions, separated by at least 5 days (appendix p 10). We chose 3 days (72 h) for the inpatient period because most food-derived metabolites are absorbed and eliminated in urine within 48 h, as evidenced in numerous studies (including other studies done in our laboratories) of the kinetics of absorption, bioavailability, and elimination of several food metabolites contributing to the urinary metabolome.^21^ The minimum 5 day gap between dietary intervention periods ensured that any possible carryover was minimised. For example, tartaric acid, a marker of grape consumption, was shown to be cleared from the body within a few hours in an excretion kinetics study.^22^ Similarly, other metabolites associated with diet, such as proline betaine, creatine, and trimethylamine N-oxide (TMAO), are cleared from the body within a few hours.^23^, ^24^

The Nutrition and Dietetic Research Group at Imperial College London (London, UK; led by GF) developed four dietary interventions with a stepwise variance in concordance with the WHO healthy eating guidelines^6^—diet 1 was the most concordant with the guidelines, and diet 4 was the least concordant (table 1; appendix p 5). High energy density is an important driver of the association between poor diets and the risk of obesity and diabetes;^25^ therefore, the diets had a range of energy densities (table 1; appendix p 5).

Participants were asked to consume all the food provided and were allowed to drink water as they wished. The expectation to consume all food provided and not to leave the CRF during each visit was fully explained to potential participants before they provided consent to take part in the study. This adherence was monitored strictly: all food was weighed immediately before being given to the participants, and any uneaten food was weighed. Physical activity was also controlled; participants were allowed to engage in only very light physical activity (no more strenuous than walking from their hospital bed to the toilet).

During each 3 day inpatient period, urine was collected daily over three timed periods: morning collection (0900–1300 h; cumulative sample 1), afternoon collection (1300–1800 h; cumulative sample 2), and evening and overnight collection (1800–0900 h; cumulative sample 3). 24 h urine samples were obtained by pooling these samples (appendix p 10). Urine samples were prepared with a pH 7·4 phosphate buffer for ^1^H-NMR spectroscopy as described previously.^26^ We analysed samples at 300 K on a 600 MHz spectrometer (Bruker BioSpin, Karlsruhe, Germany) using a standard one-dimensional pulse sequence with water-presaturation.^26^ Acquisition parameters are shown in the appendix (pp 2–3).

### Statistical analysis

To the best of our knowledge, this study was the first-in-human trial of metabolic profiling in a controlled feeding setting; therefore, no formal power calculation could be undertaken. However, to provide a basis for sample size calculation, we used data on urinary proline betaine, which we selected as a representative marker for nutritional intake.^23^ Heinzmann and colleagues^23^ suggested that urinary concentration of this metabolite would rise by 50 μmol/L with each incremental rise in fruit intake (ie, pieces of fruit) in the experimental setting. With an SD of 40 μmol/L, assuming a power of 0·95 and an alpha of 0·05 to detect a difference of 50 μmol/L, we estimated that we would need 12 volunteers. Because the protocol required a high amount of volunteer time and involvement (12 inpatient days plus travelling time) and volunteers could withdraw from the study, we requested permission to recruit 30 people, with the aim of having a cohort of roughly 20 people. Of note, in two previous dietary studies^27^, ^28^ researchers identified individual biomarkers of food intake after controlled feeding having included fewer than 20 participants in each study. All 19 participants who completed the study were included in the analysis.

^1^H-NMR spectra (16 000 spectral variables) were manually phased and digitised over the range δ0·5–9·5 and imported into MATLAB (release 2014a). A combination of data-driven^29^ and experimental structural elucidation techniques and spiking-in of chemical standards was used to aid structural identification of diet-discriminatory metabolites. We used global urinary ^1^H-NMR spectral profiles representing diets 1 and 4 to generate representative metabolite patterns relating to each diet. Global metabolic profiling entails using methods that aim to measure all metabolites, or as many as possible with the assay, in a sample, as opposed to targeted analysis, in which only specific compounds are measured. Because this study is the first of its kind, we did not know a priori which compounds were of interest; therefore, we used global metabolic profiling to capture as much information as possible rather than limit our information to a set of targeted compounds.

We modelled data with partial least squares discriminant analysis (PLS-DA), using Monte Carlo cross-validation (MCCV) to assess model robustness using a total of 1000 individual models; the data were centred and scaled to account for the repeated-measures design. The mean (T_pred_) and variance of each predicted sample were estimated using all MCCV models. We then used this MCCV–PLS-DA model to predict 24 h urinary global profiles of volunteers after 3 days of strict adherence to the intermediate diets (ie, diets 2 and 3) without informing the model whether these urinary profiles belonged to diet 2 or diet 3. Day 3 samples were used for modelling because these were timed to be 48 h after starting the dietary intervention and ensured diet homoeostasis. Data from day 1 and day 2 samples served as internal validation data.

Across the 1000 models the mean prediction (T_pred_) of each sample was calculated from all models in which the sample was part of the validation set. A positive T_pred_ indicates that the urinary metabolic profile of the sample resembles more diet 1 than diet 4, and vice versa for a negative T_pred_. The variance of T_pred_ was estimated from the same predictions as were used for calculating the mean. We then calculated the Kernel density estimate by summing the resulting Gaussian distributions of all samples within each group. A p value was calculated for each variable on the basis of 25 bootstrap resamplings of the training data in each of 1000 models to estimate the variance and the mean coefficient across the 1000 models. Spectral variable importance was assessed with the false discovery rate q value, with a value of 0·01 or less as the cutoff for significance.

To assess the ability of our model—based on the 24 h urinary collections—to independently predict healthy eating in a free-living population, we used data from the UK cohort (n=225 from a cohort of 499) of the INTERMAP study as our validation dataset. The INTERMAP study^30^ investigated dietary and other factors associated with blood pressure in 4680 men and women aged 40–59 years from 17 population samples in four countries (China, Japan, UK, and USA). Dietary intake data were obtained from two consecutive multipass 24 h recalls^31^ on two occasions that were 3 weeks apart on average. For this analysis, we used the 24 h urine sample data, corresponding to the first two multipass 24 h dietary recalls, from the UK cohort.^32^ We stratified participants into percentile groups (0 to 10th, 45th to 55th, and 90th to 100th) using the Dietary Approaches to Stop Hypertension (DASH) index (appendix p 2),^33^ which is a tool used for healthy eating assessment in several countries^34^ and has been used in INTERMAP.

Additionally, to assess the ability of our model to inform a non-UK dataset, we used data from a healthy omnivorous cohort of 66 participants recruited and phenotyped at the University of Copenhagen (Copenhagen, Denmark) for our external validation dataset (appendix p 7). We calculated DASH scores for these participants on the basis of their 4 day dietary records according to the quintiles defined in the appendix (pp 2, 6). In this cohort, metabolite profiling was done on spot urine samples collected after the first morning void following a 10 h overnight fast. Therefore, we mapped these samples to models derived from cumulative sample 1 (morning collection) in our trial. The method used to model spot urine samples is provided in the appendix (pp 4, 14).

To account for differences in urine osmolality, we normalised all spectra from our study cohort and the two validation cohorts using Probabilistic Quotient Normalization^35^ to the median spectrum of diets 1 and 4 combined. This procedure corrects the metabolite concentrations for differences in dilution across samples. Such differences can arise from different intakes of water or liquids between participants (causing differences in metabolite concentrations) and from different amounts of foods consumed (eg, high caloric intakes). Therefore, any effect of these potential confounders is attenuated by the normalisation procedure.

We used the Skillings-Mack and Kruskal-Wallis tests, as appropriate, to assess differences among multiple groups, and non-parametric post-hoc (Wilcoxon's signed rank and rank sum) tests to determine pairwise differences. p values from post-hoc tests were adjusted for multiple testing with Hommel's adjustment. More details on the statistical analysis are given in the appendix (pp 3–4). All statistical analyses were done in MATLAB.

This trial was registered on the NIHR UK clinical trial gateway and with ISRCTN, number ISRCTN43087333.

### Role of the funding source

The funders of the study had no role in study design, data collection, data analysis, data interpretation, or writing of the report. IG-P, JMP, EH, and GF had full access to all the data in the study, and the corresponding authors (GF and EH) had final responsibility for the decision to submit for publication.

## Results

**Figure 1:**
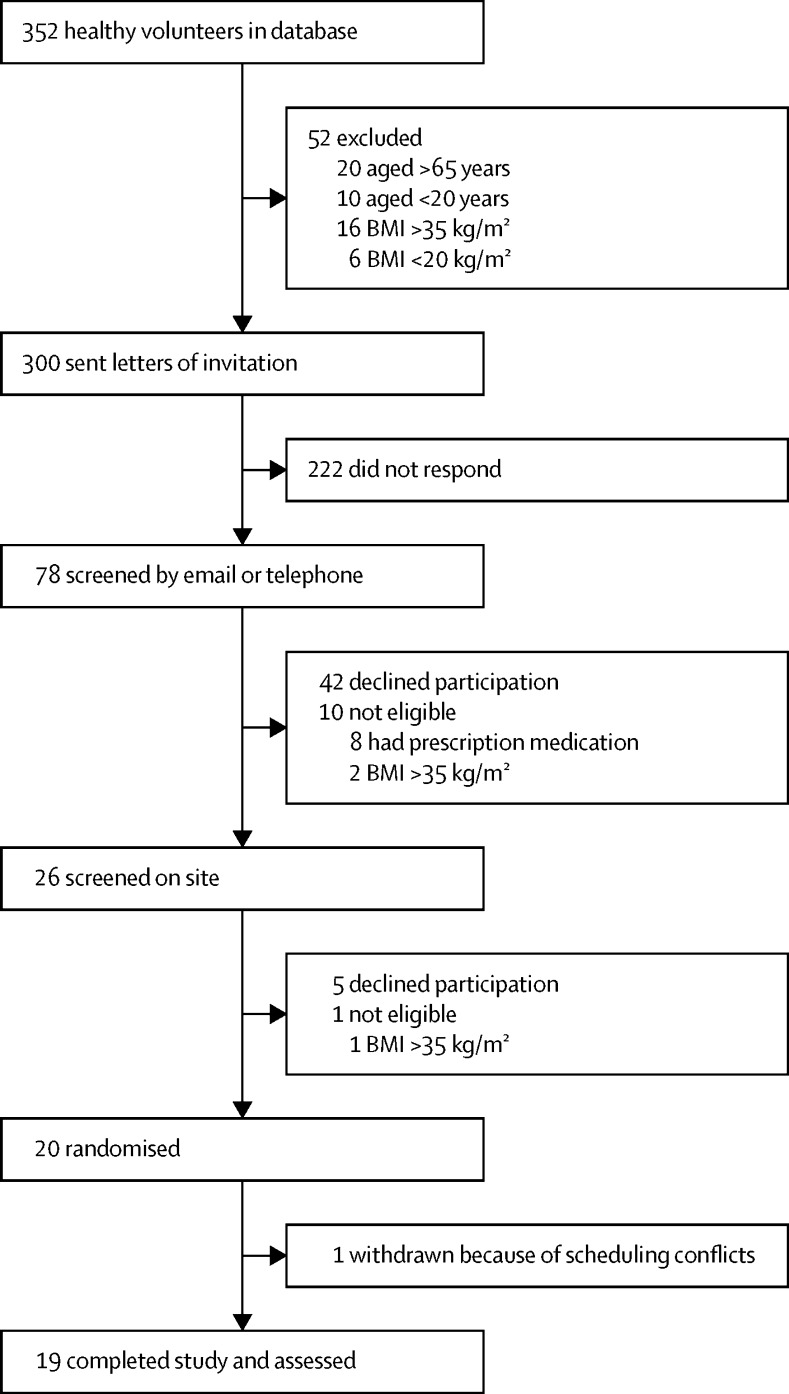
Trial profile

Of 352 individuals in the database of healthy volunteers at the NIHR/Wellcome Trust Imperial CRF, we contacted 300 who were eligible for the study with a letter of invitation between Aug 13, 2013, and May 18, 2014 (52 were ineligible on the basis of age or BMI). 78 individuals responded to the invitation and, after screening, 20 remained eligible and enrolled into the study (figure 1). Between Oct 2, 2013, and July 29, 2014, 19 participants completed the four inpatient periods and consumed all the food provided; their baseline characteristics are shown in table 2.

**Figure 2:**
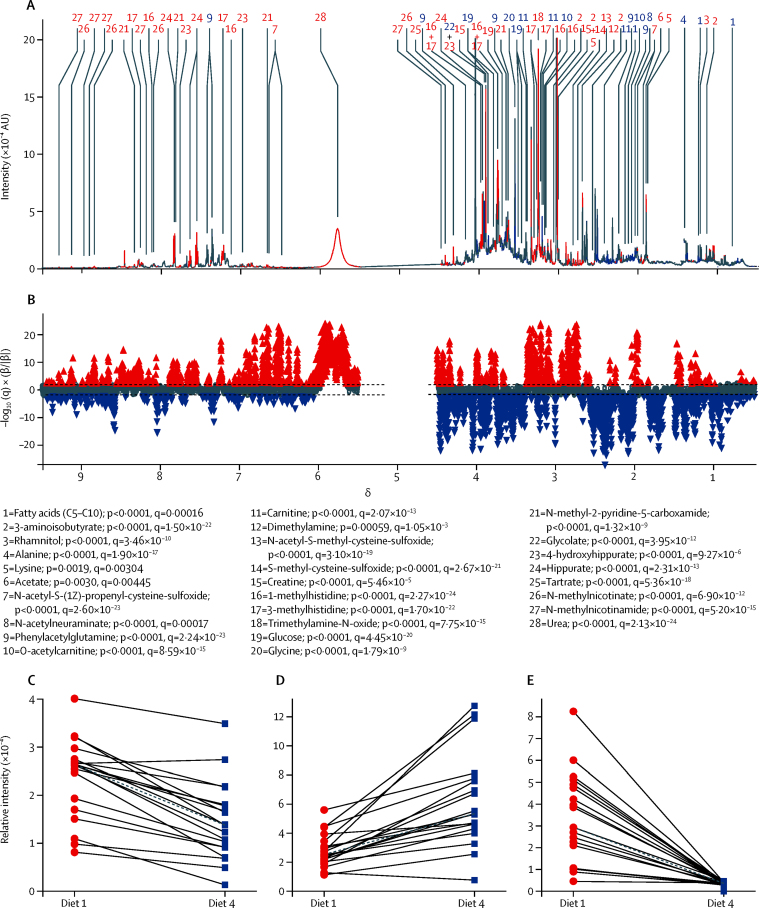
Associations of urinary metabolites with diets 1 and 4 in 19 participants Data from the third 24 h urine collection are shown; data from the first and second 24 h urine collection are shown in the appendix (p 11). (A) Mean 600 MHz ^1^H-NMR spectrum of the 19 participants. (B) Manhattan plot showing −log_10_(q) × sign of regression coefficient (β) of the MCCV–PLS-DA model for the 16 000 spectral variables. A p value was calculated for each variable on the basis of 25 bootstrap resamplings of the training data in each of 1000 models to estimate the variance. Red peaks represent the 19 metabolites excreted in higher amounts after diet 1 and blue peaks represent the nine metabolites excreted in higher amounts after diet 4. The two horizontal lines indicate the cutoffs for the false discovery rate on the log_10_ scale. (C–E) Metabolite concentrations for 19 participants after following diet 1 and diet 4 for (C) hippurate (a urinary marker of fruit and vegetable consumption; number 24 in part A), (D) carnitine (a marker of red meat consumption; number 11 in part A), and (E) tartrate (a marker of grape intake; number 25 in part A). ^1^H-NMR=proton nuclear magnetic resonance. AU=arbitrary unit. MCCV=Monte Carlo cross-validation. PLS-DA=partial least squares discriminant analysis.

**Figure 3:**
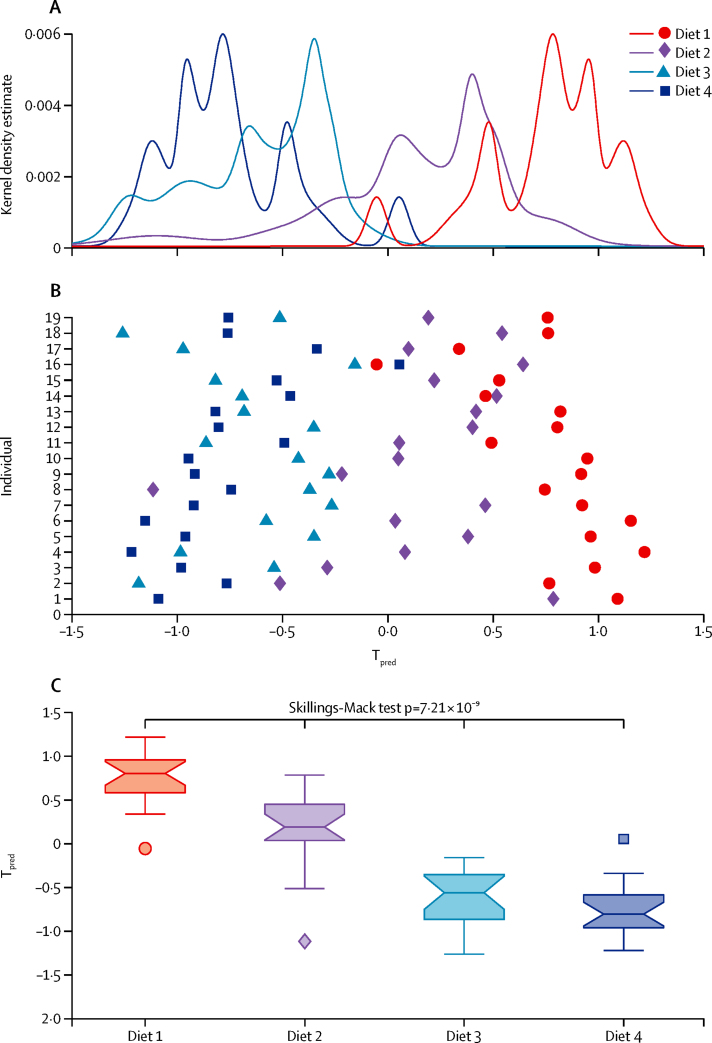
The MCCV–PLS-DA model of metabolic patterns of the four diets for 19 participants Data from the third 24 h urine collection. (A) Kernel density estimate of the predicted scores (T_pred_) for the four diets. (B) Mean predicted score for individuals' spectra after following the diets. (C) T_pred_ of the four diets. Box and whiskers plots indicate median with 25th and 75th percentiles (boxes), interval endpoints (notches of boxes), and 1·5 × IQR above or below the 75th and 25th percentiles (whiskers); points are outliers. Post-hoc Wilcoxon's signed rank test for pairwise differences (adjusted for multiple testing with Hommel's method) gave the following p values: diet 1 *vs* diet 2 p=6·71 × 10^−4^; diet 2 *vs* diet 3 p=5·04 × 10^−4^; diet 3 *vs* diet 4 p=1·96 × 10^−1^; diet 1 *vs* diet 3 p=3·05 × 10^−5^; diet 2 *vs* diet 4 p=4·58 × 10^−5^; diet 1 *vs* diet 4 p=3·05 × 10^−5^. MCCV=Monte Carlo cross-validation. PLS-DA=partial least squares discriminant analysis.

MCCV–PLS-DA models of 24 h urine spectra showed systematic differences between metabolic phenotypes of diets 1 and 4 that were reflected in both the metabolic profile (figure 2) and predicted scores (figure 3). From a total of 486 peaks (appendix p 3) in the mean ^1^H-NMR spectrum, 19 identified metabolites were present in significantly higher concentrations in urine after consumption of diet 1 than after diet 4, and nine metabolites were present in significantly increased concentrations after consumption of diet 4 (figure 2B; appendix p 8). For example, all 19 individuals had consistent, significant changes in excretion of 28 metabolites (figure 2B), including metabolites with well known dietary associations—hippurate (a urinary marker of fruit and vegetable intake), carnitine (a marker of red meat consumption), and tartrate (a marker of grape intake; figure 2C–E). Substantial between-person variability could be seen in concentrations of hippurate (figure 2C) and carnitine (figure 2D), but the direction of association remained the same. For tartaric acid, between-person variability was also apparent, but because it is a quantitative biomarker of grape consumption^22^ there was almost no between-person variation after consumption of diet 4, which did not contain any grape-derived products (appendix p 5).

Data from the MCCV–PLSA-DA model obtained from the analysis of 24 h urine samples from diets 1 and 4 were used to predict 24 h global urinary metabolic profiles of diets 2 and 3 (figure 3A). We found a significant stepwise increase in predicted scores from diet 4 (negative scores) to diet 1 (positive scores; Skillings-Mack test p=7·21 × 10^−9^; figure 3B). The urinary metabolic profiles generated from the urine samples obtained after diet 3 clustered next to those obtained after diet 4, and those from diet 2 clustered adjacent to those from diet 1. Additionally, these metabolite patterns were reproducible in 24 h samples obtained from the 19 volunteers on days 1 and 2 of the 72 h inpatient stay (appendix p 11).

**Figure 4:**
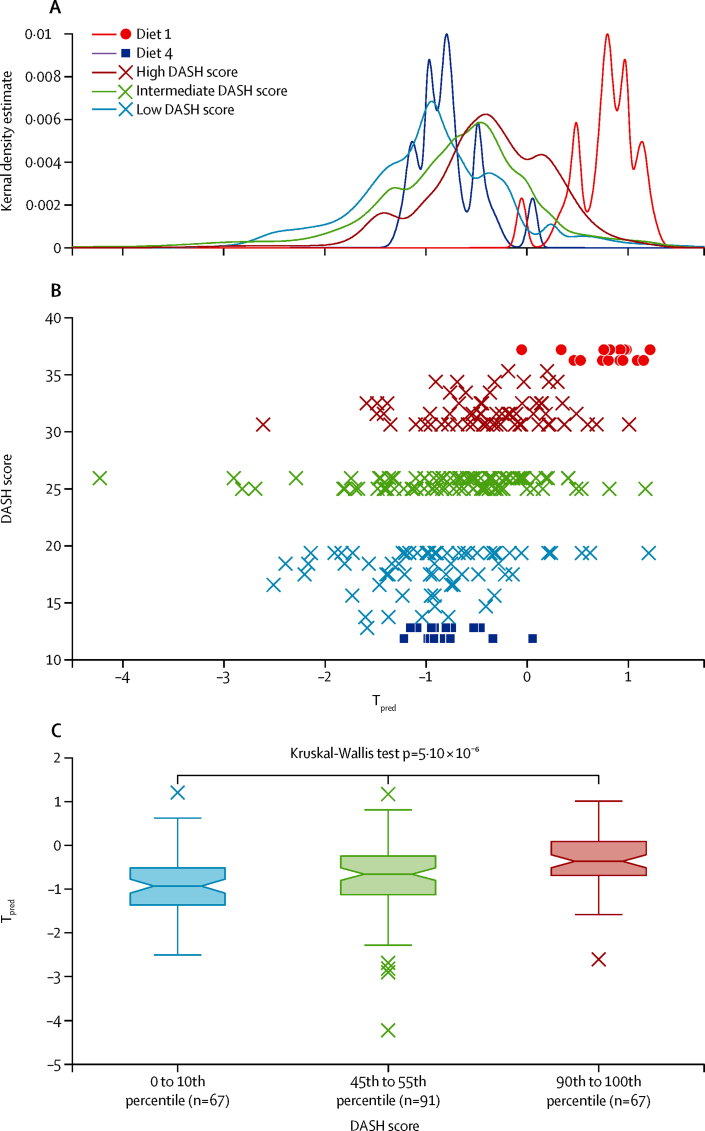
Applicability of our model to predict adherence to diverse diets in the INTERMAP UK cohort (A, B) Kernel density estimates of the predicted scores (T_pred_) of diet 1, diet 4, and the INTERMAP UK cohort stratified by DASH scores. Dots and squares represent participants from the study cohort, and crosses represent individuals from the INTERMAP UK validation cohort. (C) T_pred_ of the INTERMAP UK cohort. Box and whiskers plots indicate median with 25th and 75th percentiles (boxes), interval endpoints (notches of boxes), and 1·5 × IQR above or below the 75th and 25th percentiles (whiskers). Crosses indicate outliers—ie, if the predicted values lie outside 1·5 times of the IQR (25th to 75th percentile), corresponding to points lying outside 2·7σ (roughly 0·993 of a normal distribution) either side of the mean. Post-hoc Wilcoxon's signed rank test for pairwise differences (adjusted for multiple testing with Hommel's method) gave the following p values: 0 to 10th percentile *vs* 45th to 55th percentile p=2·32 × 10^−2^; 45th to 55th percentile *vs* 90th to 100th percentile p=4·31 × 10^−3^; 0 to 10th percentile *vs* 90th to 100th percentile p=3·53 × 10^−6^. DASH=Dietary Approaches to Stop Hypertension.

In our first validation dataset (INTERMAP UK cohort), urinary metabolic profiles of samples from the group with high DASH score (90th to 100th percentile; n=67) clustered near those from the diet 1 samples, whereas metabolic profiles of samples from the group with low DASH score (0 to 10th percentile; n=67) clustered next to those from the diet 4 samples (figure 4A). The urinary metabolic profiles from the group with intermediate DASH scores (45th to 55th percentile; n=91) clustered between the two extreme categories. Although the Kernel density estimate plot showed some overlap between the three groups—which was expected because of dietary misreporting, estimated to be 22·4% from men and 30·9% from women in the INTERMAP UK cohort^36^—a significant linear association was seen between DASH scores and predicted scores (Kruskal-Wallis test p=5·10 × 10^−6^; figure 4B). Post-hoc Wilcoxon rank sum test corrected by Hommel's method confirmed that all pairwise comparisons differed significantly (figure 4). In addition to global metabolite profiles, we quantified specific metabolites from the model known to be associated with foods linked to healthy eating—ie, hippurate (fruits and vegetables), 4-hydroxyhippurate (fruits), and S-methyl-L-cysteine-sulfoxide (cruciferous vegetables)—and found that these metabolites were present in significantly higher concentrations in urine samples from INTERMAP participants with high DASH scores than in samples from those with low DASH scores (appendix p 9). However, S-methyl-L-cysteine-sulfoxide (p=0·19) and hippurate (p=0·051) concentrations did not differ significantly between the groups with intermediate DASH scores and high DASH scores; similarly; concentrations of hippurate (p=0·096) and 4-hydroxyhippurate (p=0·15) did not differ significantly between the groups with low DASH scores and intermediate DASH scores. The overall findings indicated that the global urinary metabolic profile measured by ^1^H-NMR spectroscopy (χ^2^ 24·37, p<0·0001) was a more accurate predictor of dietary patterns than single markers of individual foods consumed (χ^2^ 12·71–21·09, p=0·0017 to p<0·0001).

We classified seven INTERMAP participants as metabolic outliers (figure 4B). On detailed examination of their dietary records, one participant (who had low DASH score but positive predicted score) was considered a misreporter because high amounts of proline betaine were found in the urine, but no citrus fruits or other dietary sources of proline betaine were recorded. The urine of another outlier from the group with intermediate DASH scores contained very high amounts of N-methylnicotinate (a vitamin B3 derivative), which contributed greatly to the classification of this sample as close to diet 1. This individual had consumed very high amounts of coffee, which is rich in niacin (vitamin B3), a precursor of N-methylnicotinate, accounting for the high level of urinary excretion. The urine of the remaining five outliers (DASH scores 24, 24, 25, 25, and 30) contained very high amounts of paracetamol, the metabolite signals (specifically sulphate and glucuronide) of which overlap with phenylacetylglutamine signals; the fact that phenylacetylglutamine was associated with diet 4 could help to explain the misclassification.

For the external validation cohort (ie, the Danish cohort; n=66), the metabolite profiles aligned with those of diets 1 and 2 (appendix p 12), which was confirmed by the high DASH score of the cohort (median 28·5, range 20–36). The urinary metabolic profiles were again associated with the dietary profiles (p<0·0001). Although our method worked best with 24 h urine models (appendix pp 11, 13), the model created for spot samples from the Danish cohort based on cumulative urine samples also showed good stratification of metabolic phenotypes according to diet.

## Discussion

In this proof-of-principle study, we showed notable differences in urinary metabolic profiles in a controlled feeding condition in which participants consumed four defined diets differing in compliance to the WHO-recommended healthy diet. We then showed, in two independent epidemiological datasets, that these metabolic profile patterns have the potential to classify the dietary intake of free-living individuals. We concluded that this novel application of metabolic phenotyping at the population level has the potential to provide objective measures of adherence to dietary recommendations, without the use of dietary surveys, which are known to be subject to misreporting, incompleteness, and bias.

We showed that urinary concentrations of biomarkers from individual healthy foods—eg, hippurate (a marker of fruit and vegetable consumption), (N-acetyl-)S-methyl-L-cysteine-sulfoxide (cruciferous vegetables), dimethylamine and TMAO (fish), and 1-methylhistidine and 3-methylhistidine (oily fish and chicken)—were significantly higher after consumption of diet 1 than after diet 4, reflecting increased intakes of fruits, cruciferous vegetables, salmon, and chicken that were provided as part of diet 1. TMAO is a cryoprotectant in freshwater and saltwater fish, and urinary concentrations of TMAO are associated with recent fish intake;^37^ therefore, high urinary TMAO concentrations can be associated with healthy diets that are rich in fish. However, gut bacteria can synthesise TMAO from choline and hence high urinary and plasma TMAO concentrations can also originate from red meat consumption, which is generally associated with adverse health outcomes. Indeed, high concentrations of TMAO in plasma and urine have been associated with cardiovascular and renal disease, respectively.^38^ Thus, the global pattern of metabolites, which reflects the totality of the diet, is more important in indicating dietary patterns than are individual biomarkers.

Although findings from previous studies have shown that metabolic profiling could be used to identify specific dietary biomarkers associated with diet, our new approach allowed differentiation of the dietary interventions on the basis of global urinary metabolic profiles, while also reflecting specific chemicals found in individual foods or beverages. We used the characteristic metabolic profiles of diets 1 and 4 to predict the dietary profiles of individuals consuming diets between these two extremes (ie, diets 2 and 3). A clear separation was seen across the metabolite profiles of the four diets. We also showed significant stepwise differences in metabolite concentrations from diet 1 to diet 4. Changes in individual diet-associated metabolites were consistent across the dietary interventions.

To assess the feasibility of using metabolic profiling as an objective and unbiased approach to assess dietary patterns in a population setting, we used the INTERMAP UK cohort as a validation dataset to investigate whether free-living individuals with high dietary DASH scores (associated with a reduced risk of non-communicable diseases) and low DASH scores (associated with a high risk of non-communicable diseases^33^, ^34^) could be distinguished by use of the metabolite profiles from the controlled feeding experiment. We showed substantial clustering of predicted scores of the metabolic profiles according to the DASH scores, suggesting that dietary patterns could be predicted by interrogating the whole urinary metabolite profile (not just individual metabolites). This approach was based on the concept that differences in dietary intake are reflected in the relative concentrations of many hundreds of urinary metabolites.^39^ Metabolic analysis revealed that urine from participants with high DASH scores in the INTERMAP cohort contained an increased abundance of biomarkers associated with fruit and vegetable intake—including hippurate, 4-hydroxyhippurate, and S-methyl-L-cysteine-sulfoxide—compared with urine from participants on a diet with low DASH scores. The dietary intervention models were able to predict dietary patterns regardless of the specific dietary components. For example, the citrus fruit marker proline betaine was present in increased abundance in samples from the group with high DASH score (appendix p 9) even though citrus fruits were not provided as part of the dietary interventions. This finding suggests that the derived metabolite profiles are reflective of a wider range of healthy diets than those used in our trial.

Although the energy intakes across the three groups in the INTERMAP cross-sectional study stratified for DASH score did not differ significantly (despite a large spread around the median), the participants with low DASH scores tended to have higher energy intakes than did those with high DASH scores (appendix p 6). However, the potential confounding effects of energy intake and dilution on the urinary metabolome were attenuated by the normalisation procedure applied.

Dietary intake data from free-living participants are subject to dietary misreporting; therefore, this challenge had to be addressed in the validation of our model. The INTERMAP epidemiological data have a track record of low misreporting rates,^36^ thus allowing us to address this limitation by exploring food intake records of so-called metabolic outliers. Because of potential misreporting issues, we used a second cohort (the Danish cohort) to further validate our model. Since only data from spot urine samples after the first void were available for this cohort, we matched the timing of this spot urine with the morning urine collection in our trial. Our analysis showed that the metabolite profiles of the Danish cohort resembled those of our participants after consumption of diets 1 and 2, and concurred with the dietary analysis showing that this healthy-eating population had a high median DASH score (appendix p 7). These findings suggest that our model is robust across different populations.

Although results from several studies^33^, ^34^ have shown that the DASH score is positively associated with health, so far the DASH score has not generally been adopted in public health policies. We based our experimental dietary interventions on internationally accepted healthy eating guidelines.^6^ Several other dietary scoring methods—such as the alternate healthy eating index^40^ and Mediterranean^41^ and alternate Mediterranean scores^42^—exist, but we chose to apply the DASH index to all the diets (the ones in our trial and the diets consumed by the INTERMAP and Danish cohorts) to provide a common scale in which the range in absolute numbers is not limited. Additionally, the DASH score has been shown to be associated with cardiac risk.^43^

It is important to understand the difference in use of spot urine samples compared with 24 h urine samples. Although spot urine samples are commonly used in epidemiological studies, they provide only the snapshot of the urinary metabolome for a very specific sampling time, whereas 24 h samples provide a time-averaged window on metabolism encompassing diurnal variation and other lifestyle-related fluctuations. Global metabolic changes are thus more difficult to assess in spot samples; use of the 24 h model to predict spot samples taken 2 h after lunch, 5 h after lunch, and 2 h after dinner showed that variability was greater in the spot sample predictions (appendix p 13) than in 24 h samples from days 1, 2, and 3 of the trial (appendix p 12). Where possible, use of 24 h urine samples rather than spot samples is advised for measurement of metabolic changes, particularly when excretion kinetics are not known a priori, partly because spot urine samples vary more in dilution. However, where only spot urine samples are available because of study limitations, such as in the case of the Danish cohort, we found that the time-matched cumulative sample obtained between meals predicted the spot samples with more accuracy than did the 24 h model (appendix p 14), which is consistent with previous work showing accurate quantification of grape intake based on both 24 h samples and cumulative samples matching the excretion kinetics window.^22^ For this reason, we compared the spot samples, taken after the first morning void in the Danish cohort with the model derived from cumulative sample 1 (after breakfast to before lunch) (appendix p 11) rather than the 24 h model, because the sampling time was better matched.

Our study has several limitations. The metabolic profiling trial and the models derived from it were based on limited types of foods. However, even with this narrow range of foods, the model clearly classified the diets from the corresponding urinary metabolic profiles. To the best of our knowledge, this study is the first of its kind to use global urinary metabolic profiles to objectively assess dietary patterns. However, we recognise that our approach will benefit from additional testing in a wider range of populations, including those of disease groups and diverse ethnic origins. Additionally, the likely misclassification of individuals consuming very high amounts of paracetamol and coffee in our model could be addressed in future work by using further analytical chemical profiling methods to avoid peak overlap in the urinary spectra. We anticipate that models built from a dataset based on a wider variety of foods would give more robust predictions. In this first-in-human study, we planned to test the hypothesis that adherence to different dietary intake patterns could be assessed from analysis of the urinary metabolome. With this aim in mind, we considered it was important that our controlled feeding study was done in healthy individuals to minimise the risk that metabolite profiles in urine might be confounded by pre-existing disease or concurrent medications, both of which are known to affect the urinary metabolic profile. The performance of the model in populations with non-communicable diseases would be an obvious follow-up. Despite these limitations, our models were predictive of dietary patterns in two well characterised free-living cohorts, thus providing proof of principle that this new approach has value as an objective means to monitor dietary patterns at the population level. Our model showed a clear separation between diets 1 and 4 in the global urinary metabolic profile and individual food-related metabolites, even though energy provided by the diets was not matched to individual estimated requirements.

Existing methods for dietary assessment—eg, dietary diaries (which require coding and data entry), food frequency questionnaires, and dietary recalls—are expensive (our own estimated cost is £60 for the complete analysis, including quality control of a 1 day dietary recall by an experienced nutritionist or dietitian). Accurate reporting assumes knowledge of food ingredients and can involve complex decoding of food product labels. Translating our study method into clinical practice is a cost-effective and time-effective alternative for objective dietary assessment—the cost is roughly £20 per sample for robust analysis by NMR, which takes less than 5 min per sample to run (excluding sample preparation). This metabolic phenotyping strategy circumvents bias caused by misreporting and the fact that dietary scoring methods, such as DASH scores, can be based on arbitrary thresholds. Moreover, similar scores can arise from different dietary patterns. The global metabolic profile, consisting of both food-specific and general food-related metabolites, is an unbiased approach and does not rely on arbitrary cutoffs. Additionally, the global metabolic profile also allows testing of whether metabolites associated with reported foods are found in the urine and therefore the accuracy of dietary records.

In conclusion, we showed that urinary metabolic profiles developed in a controlled environment have the potential to be used to assess adherence to dietary patterns in free-living populations without the need to collect dietary data. The extension and application of this strategy at the population level offer a potential step-change for public health nutrition because it provides an objective method to survey dietary intakes. Implementation of this strategy for urine-based dietary pattern analysis might enhance our understanding of the relation between diet and health and might improve clinical nutrition practice by providing health professionals with objective information on adherence to healthy eating guidelines.

## Figures and Tables

**Table 1:** Macronutrient content and characteristics of the dietary interventions

	**Diet 1**	**Diet 2**	**Diet 3**	**Diet 4**
Energy (kcal)	2260	2259	2427	2490
Energy density (kcal/g)	1·2	1·5	1·6	1·9
Proportion of protein	24%	22%	16%	13%
Proportion of carbohydrate	51%	51%	46%	44%
Total sugar (g)	14	18	22	25
Proportion of fat	23%	24%	35%	42%
Saturated fatty acids (g)	5	7	19	20
Monounsaturated fatty acids (g)	8	6	14	12
Polyunsaturated fatty acids (g)	8	5	4	2
Total trans fatty acids (g)	0·5	0·5	1	1
Fibre (g)	45·9	32·1	31·5	13·6
Sodium (mg)	2367	2261	3812	3066
Fruit and vegetables (g)	600	300	180	100
DASH score	37	30	24	11

Specific diet information (foods consumed at specific times) is shown in the appendix (p 5). DASH=Dietary Approaches to Stop Hypertension.

**Table 2:** Baseline characteristics

		**Data (n=19)**
Sex		
	Male	10 (53%)
	Female	9 (47%)
Age (years)		55·8 (12·6; 29–65)
Ethnic origin		
	White	18 (95%)
	Asian	1 (5%)
Weight (kg)		74·5 (12·5; 52·8–107·9)
BMI (kg/m^2^)		25·6 (3·2; 21·1–33·3)
Energy expenditure (kcal/day)*		2099 (351; 1668–2995)
Glucose (mmol/L)†		4·8 (0·4; 4·1–5·4)
HbA_1c_ (%)†		5·5% (0·1, 5·1–5·8)
HbA_1c_ (mmol/mol)†		36·4 (0·9; 32–40)
Triglycerides (mmol/L)‡		0·9 (0·3; 0·5–1·4)
Cholesterol (mmol/L)‡		
	Total	5·1 (0·7; 3·9–6·1)
	LDL	3·1 (0·7; 1·7–4·2)
	HDL	1·6 (0·4; 0·9–2·6)
Liver function tests (IU/L)‡		
	Alanine transaminase	21·2 (7·4; 12·3–40·0)
	Aspartate transaminase	19·5 (3·2; 15·0–24·3)

Data are n (%) or mean (SD; range). IU=international units.

* Estimated with a physical activity correction of 1·4 in all participants (appendix p 2).

† From plasma samples.

‡ From serum samples.
