# Supplementary material for: Objective assessment of dietary patterns by use of metabolic phenotyping: a randomised, controlled, crossover trial
Source: Lancet Diabetes Endocrinol. 2017 Mar;5(3):184–95. doi: 10.1016/S2213-8587(16)30419-3 (PMC5357736; doi:10.1016/S2213-8587(16)30419-3)
Supplement: Supplementary appendix [file mmc1.pdf]

THE LANCET
Diabetes & Endocrinology

Supplementary appendix

This appendix formed part of the original submission and has been peer reviewed. We post it as supplied by the authors.

Supplement to: Garcia-Perez I, Posma JM, Gibson R, et al. Objective assessment of dietary patterns by use of metabolic phenotyping: a randomised, controlled, crossover trial. *Lancet Diabetes Endocrinol* 2017; published online Jan 11. http://dx.doi.org/10.1016/S2213-8587(16)30419-3.

Appendix

Title

Objective assessment of dietary patterns using metabolic phenotyping: a randomized, controlled, crossover trial

Authors

Isabel Garcia-Perez*, Joram M Posma*, Rachel Gibson, Edward S Chambers, Tue H Hansen, Henrik Vestergaard, Torben Hansen, Manfred Beckmann, Oluf Pedersen, Paul Elliott, Jeremiah Stamler, Jeremy K Nicholson, John Draper, John C Mathers, Elaine Holmes* and Gary Frost*

Nutrition and Dietetic Research Group, Division of Endocrinology and Metabolism, Department of Medicine, Imperial College London (I Garcia-Perez PhD, R Gibson BSc, E S Chambers PhD, Prof G Frost PhD)
Biomolecular Medicine, Division of Computational and Systems Medicine, Department of Surgery and Cancer, Imperial College London (I Garcia-Perez PhD, J M Posma PhD, Prof J K Nicholson PhD, Prof E Holmes PhD)
The Novo Nordisk Foundation Center for Basic Metabolic Research, Section of Metabolic Genetics, Faculty of Health Sciences, University of Copenhagen, Denmark (T H Hansen MD, H Vestergaard MD, Prof T Hansen MD, Prof O Pedersen MD)
Faculty of Health Sciences, University of Southern Denmark, Odense, Denmark (Prof T Hansen PhD)
Institute of Biological, Environmental and Rural Sciences, Aberystwyth University UK (M Beckmann PhD, Prof J Draper PhD)
Department of Epidemiology and Biostatistics, Medical Research Council-Public Health England (MRC-PHE) Centre for Environment and Health, School of Public Health, Imperial College London, London UK (Prof P Elliott PhD)
MRC-NIHR National Phenome Centre, Department of Surgery and Cancer, Imperial College London, UK (Prof Paul Elliott PhD, Prof J K Nicholson PhD, Prof E Holmes PhD)
Department of Preventive Medicine, Feinberg School of Medicine, Northwestern University, Chicago, USA (Prof J Stamler MD)
Human Nutrition Research Centre, Institute of Cellular Medicine, Newcastle University, UK (Prof J C Mathers PhD)

* These authors contributed equally. Correspondence to Prof Elaine Holmes and Prof Gary Frost.

Correspondence to: Prof Gary Frost; Head of the Nutrition and Dietetic Research Group, Division of Diabetes, Endocrinology and Metabolism, 6th Floor Commonwealth Building, Faculty of Medicine, Imperial College Hammersmith Campus, DuCane Road, London W12 ONN. Tel.: +44 (0) 20 8383 8037. Prof Elaine Holmes, Head of Division of Computational and Systems Medicine, Department of Surgery and Cancer, Sir Alexander Fleming Building, Faculty of Medicine, Imperial College South Kensington Campus, Exhibition Road, London SW7 2AZ. Tel.: +44 (0) 20 7594 3220.
Email: g.frost@imperial.ac.uk and elaine.holmes@imperial.ac.uk.

Content

Supplementary Methods (pages 2-4)
Supplementary Tables (pages 5-9, 4 tables)
Supplementary Figures (pages 10-14, 5 figures)
Supplementary References (pages 15-16)
Supplementary Protocol (pages 17-32)

Supplementary Methods

External validation data (1) – The INTERMAP study investigates dietary and other factors associated with blood pressure[1] (BP). INTERMAP surveyed a total of 4,680 men and women aged 40-59 from 17 population samples in four countries (People's Republic of China, Japan, United Kingdom, and United States) at two time-points ('visits'). Dietary intake data were collected at each visit from 24-hour recalls conducted by trained interviewers[2]. In this study, first visit data from the two U.K. population samples (n=499) were used to study the extrapolation of results from the controlled clinical trial data. The Dietary Approaches to Stop Hypertension (DASH) index was used to stratify the INTERMAP UK data into percentile groups. The DASH score from Fung *et al*.[3] was applied to the INTERMAP UK cohort as it uses estimated servings of seven food groups (whole grains, low fat dairy, nuts and legumes, fruits, vegetables excluding potatoes, processed meat and sugar sweetened beverages) and sodium to derive the score. To allow calculation of food group intake data, composite dishes (i.e. dishes made from ingredients from two or more food groups, e.g. chilli con carne) were disaggregated following previous methodology[4, 5]. Gender specific quintiles of intake for each food group (g/day) and sodium (mg/day) were calculated and points allocated to participants. Positive scoring (quintile 1 = 1 point, …, quintile 5 = 5 points) was applied to whole grains, low fat dairy, nuts and legumes, fruits, vegetables excluding potatoes, and negative scoring (quintile 1 = 5 point, …, quintile 5 = 1 point) was applied to processed meat, sugar sweetened beverages and sodium. Higher scores signify a "healthier" diet. The quintile cut-offs are listed in **Supplementary Table 3A**. To create a range for healthy eating intakes three groups were extracted from this cohort to represent unhealthy dietary intake (DASH-scores 0–10 percentile, n=67), mid-range healthy intakes (DASH scores 45–55 percentile, n=91) and healthy dietary intakes (DASH-scores (90–100 percentile, n=67). The resulting population characteristics are summarized in **Supplementary Table 3B**. The estimated energy expenditure[6] was calculated using a physical activity correction of 1·4 in all participants.

External validation data (2) – A healthy cohort of 66 participants from Denmark was used as validation data set and DASH scores were calculated based on the INTERMAP U.K. quintiles. For this study, spot urine samples were collected after the first morning void to test the applicability of the model to different types of samples.

NMR spectroscopy and data pre-processing – The NMR analysis was performed at 300K on a Bruker 600MHz spectrometer using the following standard one-dimensional pulse sequence with saturation of the water resonance: RD – $g_{z,1}$ – 90° – t – 90° – t_m – $g_{z,2}$ – 90° – ACQ, where RD is the relaxation delay, t is a short delay typically of about 4μs, 90° represents a 90° radio-frequency pulse, t_m is the mixing time (10ms), $g_{z,1}$ and $g_{z,2}$ are magnetic field z-gradients both applied for 1ms, and ACQ is the data acquisition period (2·7s). Water suppression was achieved through continuous wave irradiation at the water resonance frequency using 25Hz radio-frequency pulse strength during the RD and t_m. The receiver gain was set to 90·5 for all experiments. Each urine spectrum was acquired using 4 dummy scans, 32 scans, 64K time domain points and with a spectral window set to 20 ppm for urine. Prior to Fourier transformation, the free induction decays were multiplied by an exponential function corresponding to a line broadening of 0·3 Hz. ^{1}H NMR spectra were manually phased and digitized over the range δ -0·5 to 9·5 and imported into MATLAB (2014a, MathWorks, Natick, U.S.A.). Each spectrum was baseline corrected. Spectra were subsequently referenced to the internal chemical shift reference (trimethylsilyl-[2,2,3,3,-$^2H_4$]-propionate, TSP) at δ 0·0. Spectral regions corresponding to the internal standard (δ -0·5 to 0·5) and water (δ 4·5 to 5·5) were

excluded. INTERMAP samples included boric acid as preservative which has been shown previously not to materially affect analytical outcome.[7]

To achieve accurate quantification of selected metabolites, resonances of each metabolite and TSP were fully relaxed using a long relaxation delays ($7 \times T_1$) between each pulse. The interpulse delay time d_1 was therefore set to 100s.[8]

Structural characterization of metabolites – A combination of data-driven and analytical identification strategies were used to aid structural identification of significant discriminatory metabolites. A total of 486 ^{1}H-NMR spectroscopic peaks were identified in the mean ^{1}H-NMR spectrum that have estimated intensities of at least 3 times intensity of the noise. Metabolites can have multiple peaks in the ^{1}H-NMR spectrum and because we use global metabolic profiling we are only interested in the variables (that make up the peaks) that are statistically significant (see below). It is for this reason we cannot give an estimation of the number of metabolites that are analysed by ^{1}H-NMR spectroscopy. SubseT Optimization by Reference Matching (STORM)[9] was used for data-driven metabolite identification of the significant ($q \leq 0 \cdot 01$) ^{1}H NMR variables. A Bruker reference library was used to assign the chemical shifts to metabolites.

Further analytical experiments were performed using a catalogue of 1D ^{1}H NMR sequence with water pre-saturation and 2D NMR experiments such as J-Resolved spectroscopy, ^{1}H–^{1}H TOtal Correlation SpectroscopY (TOCSY), ^{1}H–^{1}H COrrelation SpectroscopY (COSY), ^{1}H–^{13}C Hetero-nuclear Single Quantum Coherence (HSQC) and ^{1}H–^{13}C Hetero-nuclear Multiple-Bond Correlation (HMBC) spectroscopy.

Solid phase extraction (SPE) chromatography coupled with NMR and mass spectrometry (MS) was carried out to identify some metabolites. All the SPE fractions were analysed by 1D ^{1}H NMR spectroscopy and MS. In addition 2D NMR experiments were run for specific SPE fractions.

Finally, metabolites were confirmed by *in situ* spiking experiments using authentic chemical standards. **Supplementary Table 3** lists all metabolites identified and their chemical shifts, these are labelled in the ^{1}H-NMR spectrum in **Figure 2**.

Statistical analysis – The controlled clinical trial data was modelled using Partial Least Squares[10] (PLS) in a Monte-Carlo cross-validation[11] (MCCV) framework. In this framework data are split into many different training and test sets to obtain a robust and reliable estimation of the regression coefficients and predicted scores. In addition, for each of 1,000 models, we estimate the variability of the regression coefficients by running an additional 25 models with bootstrap resampling[12] from the training data. The 1,000 models give a robust estimate of the mean regression coefficients and the 25,000 bootstrap models can be used to estimate the variance. Together these yield a t-score for each variable and subsequently *P*-values, these *P*-values are then adjusted for multiple testing by calculating the False Discovery Rate (FDR, q-value)[13]. This can give an estimate of the consistently (same mean, small variance) and similarly (same sign) contributing variables that are the most robust contributors in the model.

Prior to the calculation of each model the data is split into training and test (validation) sets, and the data is mean-centered within-person to account for the repeated measures design by subtracting the mean of the two spectra from the spectra of each individual, this removes between-person offsets from the data. Next, the standard deviations of the variables using the training set data are calculated, and subsequently each variable from both data sets (training and test) are divided by the standard deviation from the training data to make all variables equally important in the model (unit variance).

The external validation cohorts were centered using the mean of the training data and scaled using the same standard deviation as for the test data. Data from other controlled clinical trial samples (e.g. cumulative sample 3 (CS3)) that are predicted by another model (e.g. 24-h) were centered using the mean of the independent test set (e.g. CS3), however scaling was done using the training set standard deviation. The repeated-measures centering of training/test samples results in symmetric (around 0) scores of the PLS Discriminant Analysis (PLS-DA) model for samples involved in the modelling, however for the matching samples from other diets the symmetry is not retained as they are scaled using the mean from the training set.

Across all 1,000 models the variance of the predicted score of each test sample can be estimated, this yields a Gaussian distribution for each test object. The Kernel Density Estimate (KDE) is calculated by summing the distributions within each group.

In order to assess differences between the multiple paired samples (diets) in the controlled clinical trial, addressing potentially missing data, Skillings-Mack tests were used. If appropriate, Wilcoxon signed rank *post hoc* tests were performed to determine which diets were significantly different from each other based on the predicted scores.

For the INTERMAP cohort a Kruskal-Wallis (non-parametric) test was performed to assess whether differences between any of the three INTERMAP DASH-score based groups are observed, and if appropriate, Wilcoxon rank sum *post hoc* tests were performed.

For the Danish cohort, Wilcoxon rank sum tests were performed to compare the predictions of the Danish cohort with the controlled clinical trial data predictions.

P-values from *post hoc* tests were adjusted for multiple testing using Hommel's adjustment[14].

Supplementary Table 1. Diet information for each of the 4 diets provided to the 19 volunteers.

Meal type (time)	Diet 1 Food	Diet 1 Amount (g)	Diet 2 Food	Diet 2 Amount (g)	Diet 3 Food	Diet 3 Amount (g)	Diet 4 Food	Diet 4 Amount (g)
**Breakfast (09:00)**	*Whole wheat cereal*	60	*Sugar coated cereal*	15	*Sugar coated cereal*	30	*Sugar coated cereal*	60
	Semi-skimmed milk	150	*Whole milk*	50	*Whole milk*	100	*Whole milk*	150
	Wholemeal bread, toasted	60	*White bread, toasted*	20	*White bread, toasted*	40	*White bread, toasted*	60
	Margarine, polyunsaturated	10	*Butter*	2·5	*Butter*	7·5	*Butter*	10
	Egg, hard boiled	60	*Whole wheat cereal*	40	*Whole wheat cereal*	20		
			Semi-skimmed milk	100	*Semi-skimmed milk*	50		
			Wholemeal bread, toasted	40	*Wholemeal bread, toasted*	20		
			Margarine, polyunsaturated	7·5	*Margarine, polyunsaturated*	2·5		
			Egg, hard boiled	30				
**Morning Snack (11:00)**	*Apple, Granny Smith*	150	*Apple, Granny Smith*	100	*Low fat yoghurt*	125	*Greek yoghurt*	125
					Apple, Granny Smith	50		
**Lunch (13:00)**	*Salmon, steamed*	150	*Cod, steamed*	150	*Sausage casserole*	125	*Pork sausages, fried*	125
	Jacket potato	200	*New potato*	200	*Oven chips, baked*	150	*Potato waffles, grilled*	120
	Garden peas, boiled	60	*Garden peas, boiled*	30	*Garden peas, boiled*	15	*Cola*	330
	Carrots, boiled	60	*Carrots, boiled*	30	*Carrots, boiled*	15		
	Broccoli, boiled	100	*Broccoli, boiled*	75	*Broccoli, boiled*	50		
			Diet cola	330	*Cola*	330		
**Afternoon Snack (15:00)**	*Grapes*	150	*Dark Chocolate*	50	*Milk Chocolate*	22·5	*Milk Chocolate*	45
			Grapes	100	*Dark Chocolate*	25		
					Grapes	50		
**Dinner (18:00)**	*Chicken breast, grilled*	125	*Chicken breast, fried*	125	*Beef burgers, grilled*	100	*Beef burgers, fried*	100
	Whole wheat pasta	150	*White pasta*	150	*Oven chips, baked*	150	*Potato waffles, grilled*	120
	Peppers	80	*Peppers*	40	*Baked beans in tomato sauce*	150	*Processed cheese*	30
	Onion	40	*Onion*	20	*Cheddar cheese*	40	*Tomatoes*	100
	Tomato pasta sauce	150	*Tomato pasta sauce*	150	*Diet Cola*	330	*Cola*	330
			Diet cola	330				
**Evening Snack (21:00)**	*Wholemeal bread, toasted*	80	*White bread, toasted*	40	*White bread, toasted*	40	*White bread, toasted*	80
	Margarine, polyunsaturated	10	*Butter*	2·5	*Butter*	7·5	*Butter*	10
			Wholemeal bread, toasted	40	*Wholemeal bread, toasted*	40		
			Margarine, polyunsaturated	7·5	*Margarine, polyunsaturated*	2·5		

Supplementary Table 2A. Descriptive characteristics for INTERMAP U.K. population. Population and dietary characteristics for the three DASH-groups in the INTERMAP population (n=225). Results are the median (range).

Descriptor	Low DASH (n=67)	Mid DASH (n=91)	High DASH (n=67)
	Median [range]		
DASH score	17 [11 – 18]	25 [24 – 25]	31 [30 – 35]
Age (years)	46 [40 – 59]	49 [40 – 59]	49 [40 – 59]
BMI (kg/m^2)	26·48 [18·86 – 50·51]	26·36 [20·17 – 39·70]	26·59 [20·24 – 38·16]
SBP (mmHg)	119 [92·5 – 177]	120 [93 – 161]	118·5 [93·5 – 190·5]
DBP (mmHg)	77 [44 – 108]	77 [55·5 – 97·5]	78 [35·5 – 112]
Energy (kCal/day)	2118 [964 – 4771]	1971 [818 – 4689]	1897 [1002 – 3619]
Protein (g/day)	79·90 [30·48 – 201·40]	83·13 [35·61 – 154·93]	73.42 [48·15 – 231·57]
Animal protein (g/day)	50·70 [15·22 – 167·31]	48·22 [10·30 – 101·64]	40·85 [14·41 – 179·92]
Vegetable protein (g/day)	27·89 [14·75 – 64·44]	31·59 [9·00 – 91·08]	32·57 [13·00 – 77·91]
Fats (g/day)	88·80 [38·34 – 194·81]	74·59 [25·43 – 174·53]	56·76 [16·86 – 165·06]
MFA (g/day)	31·54 [12·03 – 72·00]	24·68 [7·32 – 60·04]	19·43 [4·72 – 65·40]
PFA (g/day)	14·03 [5·10 – 39·48]	15·31 [3·39 – 46·02]	12·14 [4·29 – 51·12]
SFA (g/day)	32·60 [9·92 – 84·23]	25·80 [7·57 – 71·98]	20·20 [5·22 – 54·28]
Cholesterol (mg/day)	281·22 [25·30 – 768·41]	209·78 [22·46 – 790·81]	158·06 [41·51 – 721·29]
Carbohydrates (g/day)	232·19 [104·85 – 506·60]	241·43 [84·25 – 659·92]	241·53 [121·46 – 493·65]
Sugars (g/day)	93·50 [14·44 – 344·44]	99·44 [31·94 – 291·84]	115·78 [38·93 – 287·16]
Fibre (g/day)	18·87 [5·99 – 46·04]	23.05 [8·49 – 59·70]	30·46 [14·51 – 74·61]
Starch (g/day)	132·91 [67·64 – 298·10]	135·02 [50·15 – 486·12]	132·01 [43·33 – 298·41]
Sodium (mg/day)	3609 [1495 – 8533]	3329 [1050 – 10091]	2785 [1074 – 7341]
Potassium (mg/day)	3037 [1058 – 5988]	3201 [1575 – 6460]	3655 [2508 – 7717]

Supplementary Table 2B. Descriptive characteristics for Danish cohort. Population and dietary characteristics for the healthy eating omnivorous Danish cohort (n=66). Results are the median (range).

**Descriptor**	**Danish cohort (n=66)**
	Median [range]
DASH score	28·5 [20 – 36]
Age (years)	30 [19 – 61]
BMI (kg/m^2)	22·03 [17·36 – 27·88]
SBP (mmHg)	119 [97·7 – 140·7]
DBP (mmHg)	74·2 [52·3 – 91·3]
Energy (kCal/day)	2282 [1066 – 4273]
Protein (g/day)	86·14 [40·86 – 210·09]
Fats (g/day)	84·71 [26·13 – 179·34]
MFA (g/day)	27·61 [10·00 – 70·48]
PFA (g/day)	12·45 [3·92 – 31·96]
SFA (g/day)	29·54 [7·50 – 70·71]
Cholesterol (mg/day)	313·89 [74·63 – 743·31]
Carbohydrates (g/day)	253·29 [137·99 – 507·04]
Sugars (g/day)	83·02 [31·01 – 228·46]
Fibre (g/day)	28·74 [11·12 – 55·39]
Starch (g/day)	87·53 [36·84 – 242·16]
Sodium (mg/day)	2467 [828 – 5131]
Potassium (mg/day)	3084 [1599 – 5274]

Supplementary Table 3. List of metabolites whose urinary excretion is associated with the difference between Diet 1 and Diet 4 (Figure 2).

Lists the metabolite name, significant chemical shift values and multiplicity, sign of association (↑ indicates higher excretion after Diet 1, ↓ indicates higher excretion after Diet 4), (if known) dietary sources, and the *P*-values (unadjusted) and Q-values (False Discovery Rate[13]). Metabolites are ordered based on chemical shift.

Number [a]	Metabolite name	Chemical shift (multiplicity) [b]	Association [c]	Dietary sources	*P*-value	Q-value
1	Fatty acids (C5–C10)	0·88 (m), 1·31 (m), 2·19 (m)	↓	Fats	$7·34×10^{-5}$	$1·55×10^{-4}$
2	3-aminoisobutyrate	1·19 (d), 2·6(m), 3·02(t), 3·09 (d)	↑		$6·22×10^{-25}$	$1·50×10^{-22}$
3	Rhamnitol	1·28 (d)	↑	Fruits	$6·81×10^{-11}$	$3·46×10^{-10}$
4	Alanine	1·48 (d)	↓		$6·95×10^{-19}$	$1·90×10^{-17}$
5	Lysine	1·73 (m), 1·91 (m), 3·02 (t)	↑		$1·92×10^{-3}$	$3·04×10^{-3}$
6	Acetate	1·93 (s)	↑		$2·97×10^{-3}$	$4·45×10^{-3}$
7	*N*-acetyl-*S*-(1Z)-propenyl-cysteine-sulfoxide	1·96 (dd), 2·03 (s), 6·49 (dq), 6·65 (dq)	↑	Vegetables	$6·85×10^{-26}$	$2·60×10^{-23}$
8	*N*–acetyl neuraminate	2·06 (s)	↓		$7·87×10^{-5}$	$1·65×10^{-4}$
9	Phenylacetylglutamine	2·11 (m), 2·27 (m), 3·67 (m), 4·19 (m), 7·36 (t), 7·43 (t)	↓		$4·34×10^{-26}$	$2·24×10^{-23}$
10	*O*-acetylcarnitine	2·15 (s), 3·19 (s)	↓	(Red) meats[15]	$7·50×10^{-16}$	$8·59×10^{-15}$
11	Carnitine	2·44 (dd), 3·23 (s), 3·43 (m)	↓	(Red) meats[15]	$2·48×10^{-14}$	$2·07×10^{-13}$
12	Dimethylamine	2·72 (s)	↑	Fish[16]	$5·90×10^{-4}$	$1·05×10^{-3}$
13	*N*-acetyl-*S*-methyl-cysteine-sulfoxide	2·78 (s)	↑	Cruciferous vegetables[17]	$5·75×10^{-21}$	$3·10×10^{-19}$
14	*S*-methyl-cysteine-sulfoxide	2·84 (s)	↑	Cruciferous vegetables[17]	$2·03×10^{-23}$	$2·67×10^{-21}$
15	Creatine	3·04 (s), 3·93 (s)	↑	(Red) meats[15, 18, 19]	$2·38×10^{-5}$	$5·46×10^{-5}$
16	1-methylhistidine	3·17 (2d), 3·22 (2d), 3·78 (s), 3·99 (dd), 7·17 (s), 8·12 (s)	↑ (shift)	Lean (white) meats[15, 18-20]	$1·26×10^{-27}$	$2·27×10^{-24}$
17	3-methylhistidine	3·25 (2d), 3·30 (2d), 3·78 (s), 3·99 (dd), 7·23 (s), 8·27 (s)	↑ (shift)	Lean (white) meats[15, 18-20]	$7·31×10^{-25}$	$1·70×10^{-22}$
18	Trimethylamine-*N*-oxide	3·27 (s)	↑	Fish, meats[15, 16]	$6·64×10^{-16}$	$7·75×10^{-15}$
19	Glucose	3·42 (m), 3·49 (m), 3·54 (dd), 3·74 (m), 3·84 (m), 3·91 (dd)	↓	Sugars	$6·35×10^{-22}$	$4·45×10^{-20}$
20	Glycine	3·57 (s)	↓		$3·87×10^{-10}$	$1·79×10^{-9}$
21	*N*-methyl-2-pyridine-5-carboxamide	3·65 (d), 6·67 (d), 7·83 (dd), 8·34 (d)	↑	Niacin (vitamin B3)	$2·80×10^{-10}$	$1·32×10^{-9}$
22	Glycolate	3·95 (s)	↓		$6·04×10^{-13}$	$3·95×10^{-12}$
23	4-hydroxyhippurate	3·95 (s), 6·97 (d), 7·76 (d)	↑	Fruits[18]	$3·58×10^{-6}$	$9·27×10^{-6}$
24	Hippurate	3·98 (d), 7·55 (t), 7·64 (t), 7·84 (d)	↑	Fruits, vegetables[18, 21]	$2·81×10^{-14}$	$2·31×10^{-13}$
25	Tartrate	4·34(s)	↑	Grapes[22]	$1·62×10^{-19}$	$5·36×10^{-18}$
26	*N*-methylnicotinate	4·44 (s), 8·10 (t), 8·84 (d), 9·11 (s)	↑	Niacin (vitamin B3)	$1·09×10^{-12}$	$6·90×10^{-12}$
27	*N*-methylnicotinamide	4·48 (s), 8·19 (t), 8·90 (d), 8·96 (d), 9·29 (s)	↑	Niacin (vitamin B3)	$4·23×10^{-16}$	$5·20×10^{-15}$
28	Urea	5·80 (broad s)	↑	Protein	$8·87×10^{-28}$	$2·13×10^{-24}$

[a] Number is related to the labels in **Figure 2**.

[b] The chemical shifts and multiplicities are listed for peaks from significantly associated metabolites. Peaks are only listed if they are in the range of the processed data (9·5–5·5 and 4·5–0·5 ppm). Multiplicity key is as follows: s – singlet, d – doublet, t – triplet, q – quartet, dd – doublet of doublets, dq – doublet of quartets, 2d – two doublets, m – (other) multiplet.

[c] 'Shift' indicates chemical shift variability of the peak, e.g. due to slight pH differences of the sample.

Supplementary Table 4. Quantification and significance testing of four established dietary biomarker metabolites associated with healthy foods across low, middle and high DASH–scores in the INTERMAP U.K. cohort. A Kruskal–Wallis test was done for each metabolite and subsequently Wilcoxon rank sum *post hoc* tests were performed to assess which groups were different with Hommel's adjustment for multiple testing. The median excreted amount (mmol/24 hours) is listed for each of the three DASH–groups.

**Quantified metabolite**	**Dietary source**	**Kruskal–Wallis**	**Low DASH**	**Mid DASH**	**High DASH**	**Wilcoxon rank sum test (*P*–value)** [a]		
		P–value	Median (mmol/24h)			Low vs. mid	Low vs. high	Mid vs. high
Hippurate	Fruits, vegetables[18, 21]	$1{\cdot}73\times10^{-3}$ †	2·15	2·92	3·30	$9{\cdot}61\times10^{-2}$	$1{\cdot}48\times10^{-3}$ †	$5{\cdot}14\times10^{-2}$
S–methyl–cysteine–sulfoxide	Cruciferous vegetables[17]	$1{\cdot}20\times10^{-4}$ †	0·05	0·06	0·07	$3{\cdot}12\times10^{-3}$ †	$1{\cdot}57\times10^{-4}$ †	$1{\cdot}88\times10^{-1}$
4–Hydroxyhippurate	Fruit[18]	$2{\cdot}64\times10^{-5}$ †	0·08	0·09	0·12	$1{\cdot}51\times10^{-1}$	$1{\cdot}05\times10^{-4}$ †	$4{\cdot}86\times10^{-4}$ †
Prolinebetaine [b]	Citrus fruits[23, 24 b]	$6{\cdot}76\times10^{-7}$ †	0·08	0·16	0·34	$1{\cdot}67\times10^{-4}$ †	$2{\cdot}52\times10^{-6}$ †	$3{\cdot}51\times10^{-2}$ †

[a] *P*–values are corrected for multiple testing using Hommel's method.
[b] Prolinebetaine was not associated in the controlled clinical trial model, as no citrus fruits were provided to the volunteers.
† Indicates statistical significance at $P \leq 0{\cdot}05$.

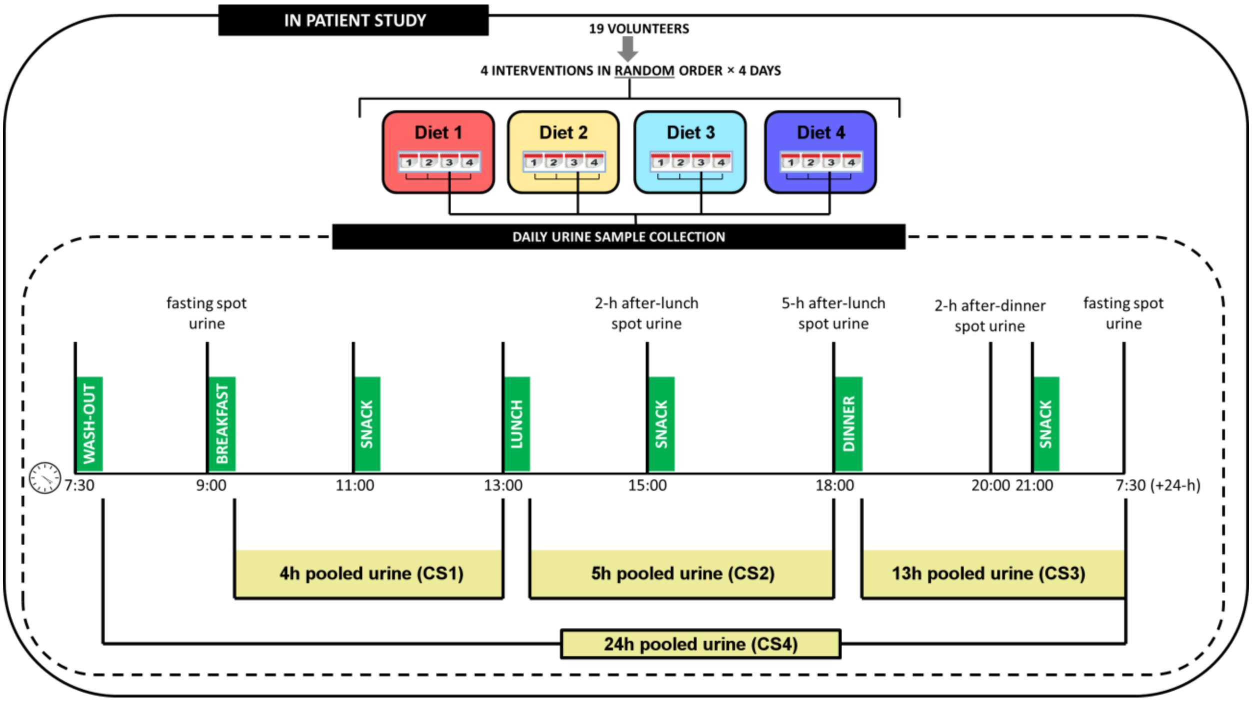

Supplementary Figure 1. Study design of the metabolic profiling study.
We used the World Health Organisation's healthy eating guidelines[25] (decrease total fat (particularly saturated fat), sugar and salt consumption and increase intakes of dietary fibre, whole grain cereals, fruit and vegetables) to develop four dietary interventions ranging from low to high metabolic risk diets. Where Diet 1 is concordant with the WHO healthy guidelines and Diet 4 is least concordant with the guidelines. 19 volunteers followed four diets for 4 consecutive days in a randomized order.

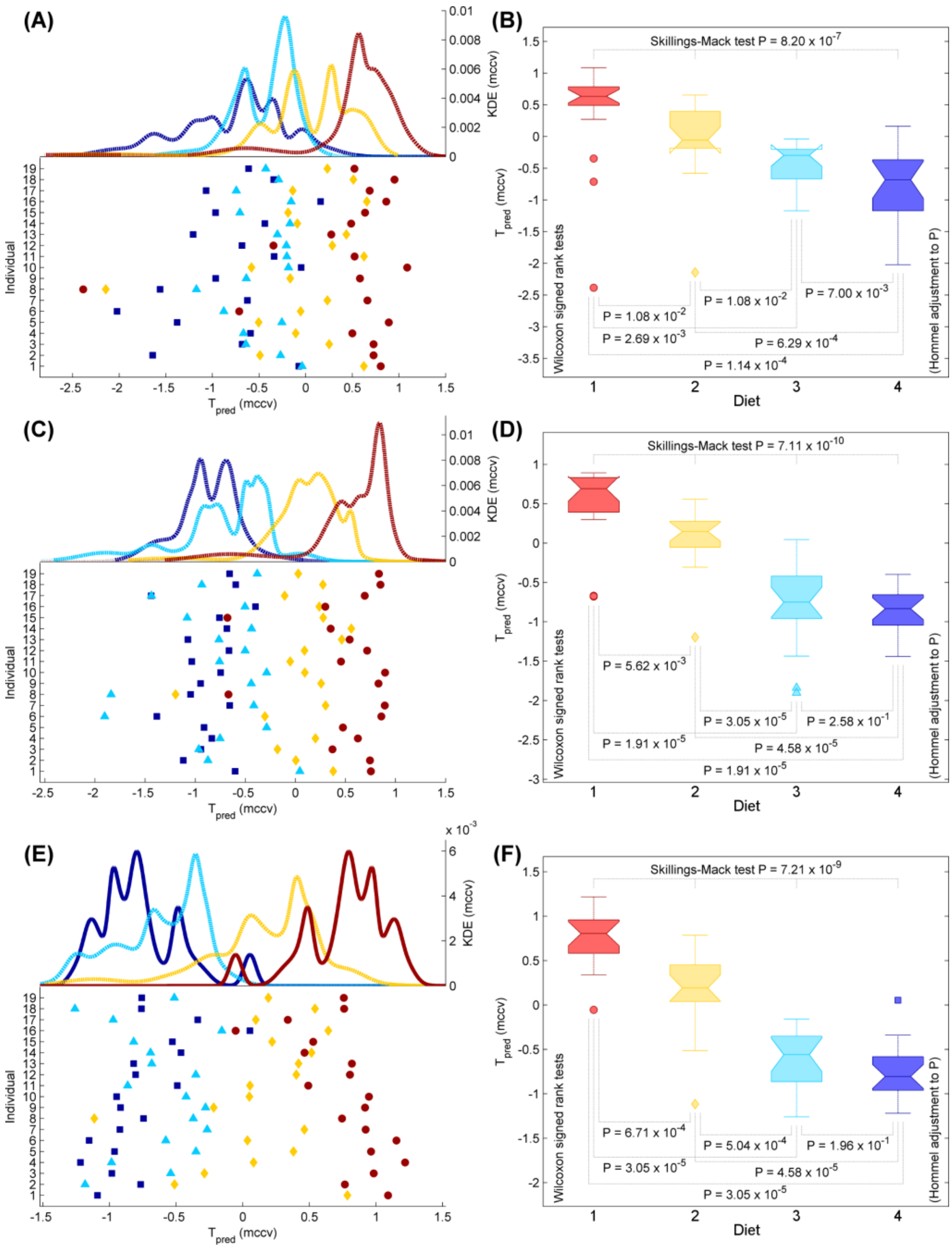

Supplementary Figure 2. Predicted scores from Monte–Carlo cross–validation (MCCV) from the 24–h urine model presented in Figure 2 of the two most extreme diets (Diet 1 and 4) predicting 24–h samples from days 1, 2 and 3 of all four diets of each individual.

(A) The data presented here is from the 24–h urine collections for day 1 predicted by the model of day 3, presented in **Figure 2**. The four DIs are represented by Diet 1 (●), Diet 2 (♦), Diet 3 (▲) and Diet 4 (■). To account for the repeated measures design the data (spectra) was mean-centered for each individual (see **Supplementary Methods**), resulting in symmetrical scores for Diets 1 and 4 for day 3. All other data are used here as validation data and thus were scaled using the mean from Diets 1 and 4 from day 3 to avoid bias. The top part of the panel gives the Kernel Density Estimate (KDE) of the predicted scores for the four diets. The bottom part shows the predicted scores (T_{pred}) from MCCV for each individual. **(B)** Boxplots of the four diets showing the predictions of the samples show linear trends between the four diets at day 1 from negative to positive based on a Skillings-Mack test and subsequent Wilcoxon signed rank tests corrected by Hommel's method. **(C)** The data presented here is from the 24–h urine collections for day 2 predicted by the model of day 3, the key is the same as in panel (A). **(D)** Boxplots of the four diets showing the predictions of the samples show linear trends between the four diets at day 2. **(E)** The data presented here is from the 24–h urine collections for day 3, also shown in **Figure 3A** and repeated here for completeness; the key is the same as in panel (A). **(F)** Boxplots of the four diets showing the predictions of the samples show linear trends between the four diets at day 3, also shown in **Figure 3B** and repeated here for completeness.

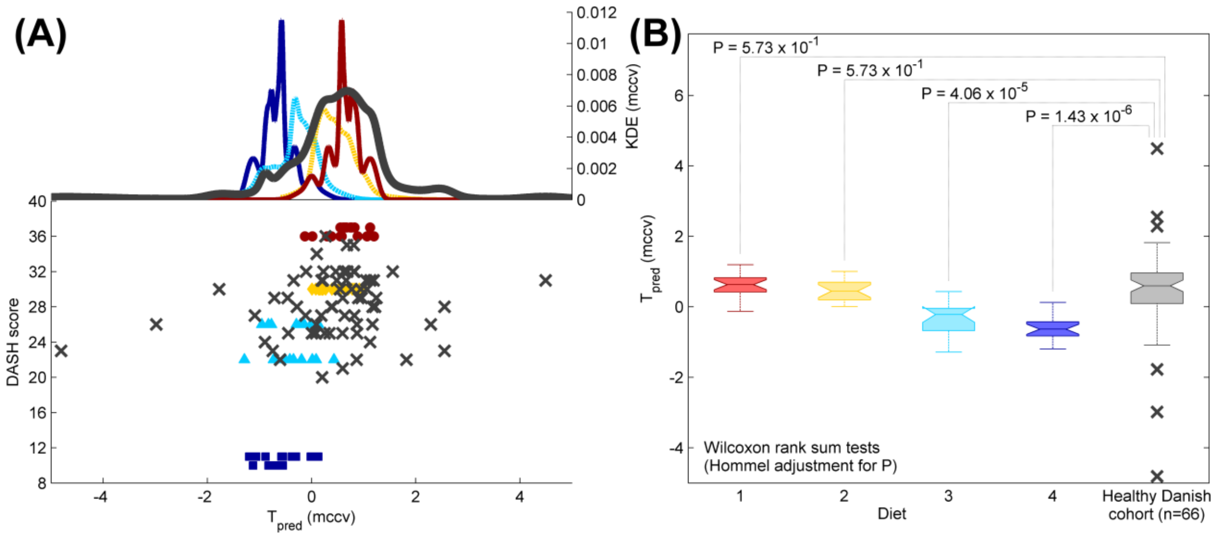

Supplementary Figure 3. The MCCV-PLS-DA model of the metabolic patterns related to the four dietary interventions and the Danish cohort's diet.

The data consist of 19 individuals in a controlled clinical trial, each individual followed each diet for three days consecutively, in randomly assigned weeks, and on each day urine was collected for 24-h. The external validation data consist of 66 healthy volunteers from the UCPH Danish omnivorous cohort. **(A)** Shows the Kernel density estimate (KDE) of the predicted scores for the four diets (top) and the average predicted score (T_{pred}, bottom) plotted against the DASH-score using quintiles calculated from the INTERMAP U.K. cohort. The data presented here are from the day-3 CS1 urine collection. Diet 1 (concordance with WHO healthy eating guidelines) is shown in red (●), Diet 2 in yellow (♦), Diet 3 in cyan (▲), Diet 4 is shown in blue (■), and the external cohort in grey (×). **(B)** Boxplot of the four dietary interventions and external validation cohort shows that the least healthy diets (Diet 4 and Diet 3) are significantly different from the external validation data based on Wilcoxon rank sum tests (*P*-values adjusted by Hommel's method) and no differences are seen between the healthy diets (Diet 1 and Diet 2) and the external validation data. A participant's score appears as outlier (×) if the predicted value lies outside 1·5×IQR (inter-quartile range) above or below the 75th and 25th percentiles, respectively. This corresponds to points lying outside ±2·7σ (≈0·993 of a normal distribution) of the mean.

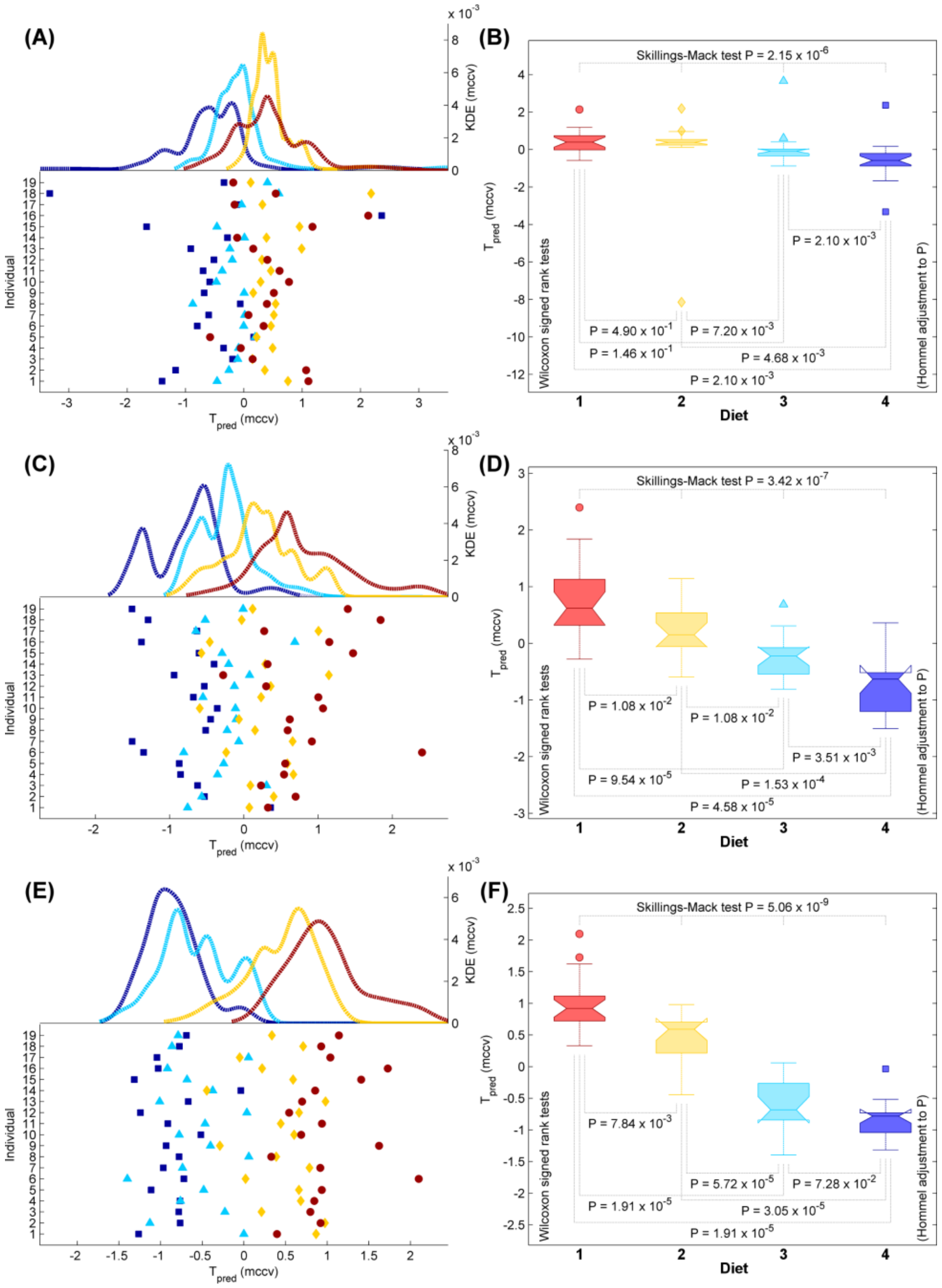

Supplementary Figure 4. Predicted scores from Monte–Carlo cross–validation (MCCV) from the 24–h urine model presented in Figure 2 of the two most extreme diets (Diet 1 and 4) predicting spot samples taken on day 3 of the DI of all four diets of each individual.

(A) The data presented here is from the 2-h after lunch spot urine samples of day 3 predicted by the model of Diet 1 and Diet 4 using 24-h urine samples, presented in **Figure 2**. The four DIs are represented by Diet 1 (●), Diet 2 (♦), Diet 3 (▲) and Diet 4 (■). To account for the repeated measures design the data (spectra) was mean-centered for each individual (see **Supplementary Methods**). All data are used here as validation data and thus were scaled using the mean from Diets 1 and 4 from day 3 to avoid bias. The top part of the panel gives the Kernel Density Estimate (KDE) of the predicted scores for the four diets. The bottom part shows the predicted scores (T_{pred}) from MCCV for each individual. **(B)** Boxplots of the four diets showing the predictions of the samples show a slight linear trend between the four diets using 2-h after lunch spot samples from negative to positive based on a Skillings-Mack test and subsequent Wilcoxon signed rank tests corrected by Hommel's method. **(C)** The data presented here is from the 5-h after lunch spot urine samples of day 3, the key is the same as in panel (A). **(D)** Boxplots of the four diets showing the predictions of the samples show stronger linear trends between the four diets for the 5-h after lunch spot samples than for the 2-h after lunch samples. **(E)** The data presented here is from the 2-h after dinner spot urine samples of day 3, the key is the same as in panel (A). **(F)** Boxplots of the four diets showing the predictions of the samples show linear trends between the four diets, the changes here are again stronger than in panels (B) and (D).

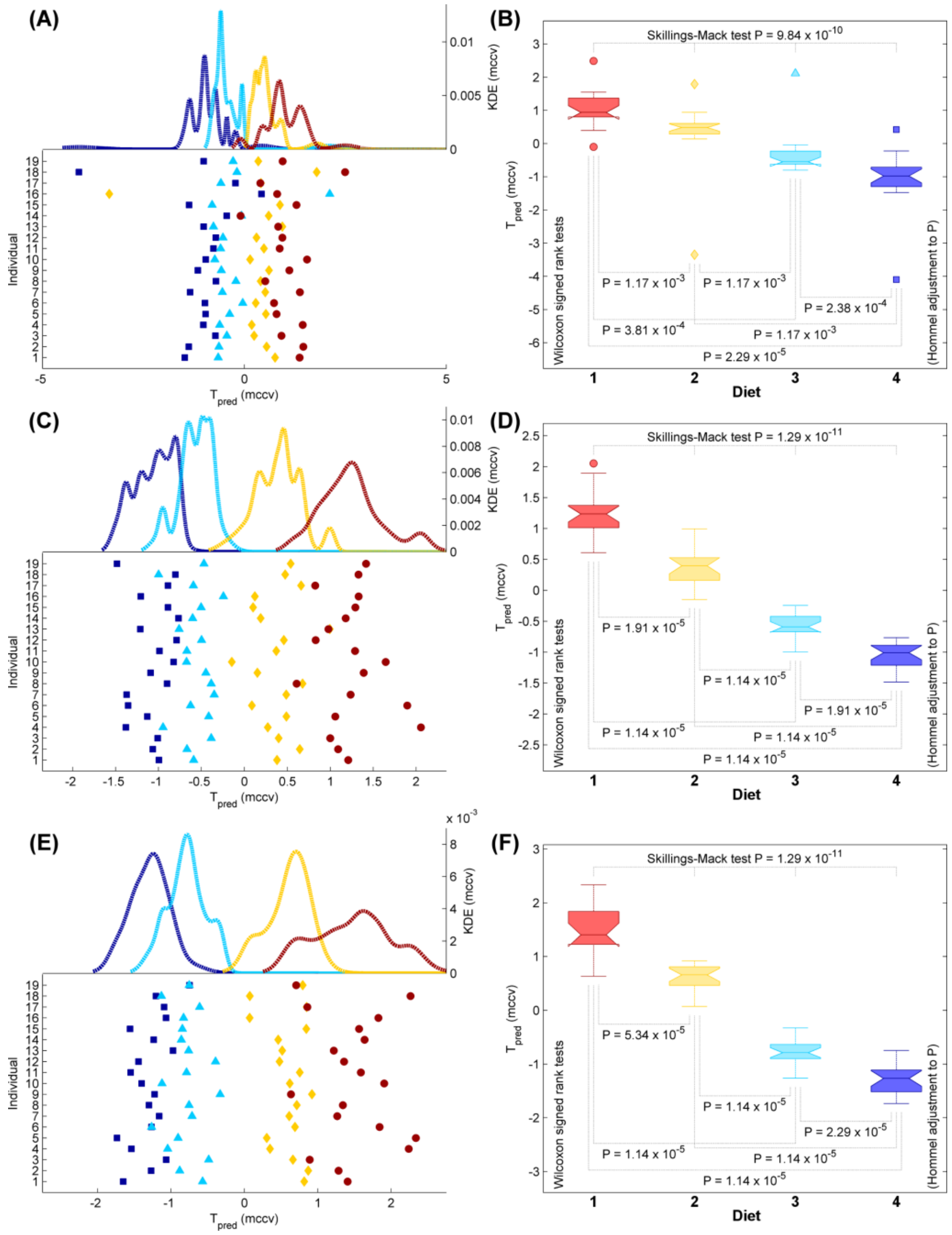

Supplementary Figure 5. Predicted scores from Monte–Carlo cross–validation (MCCV) from CS2 and CS3 models of the two most extreme diets (Diet 1 and 4) predicting spot samples taken on day 3 of the DI of all four diets of each individual.

(A) The data presented here is from the 2-h after lunch spot urine samples of day 3 predicted by the model of Diet 1 and Diet 4 using CS2 urine samples from day 3. The four DIs are represented by Diet 1 (●), Diet 2 (♦), Diet 3 (▲) and Diet 4 (■). To account for the repeated measures design the data (spectra) was mean-centered for each individual (see **Supplementary Methods**). All data are used here as validation data and thus were scaled using the mean from Diets 1 and 4 from day 3 to avoid bias. The top part of the panel gives the Kernel Density Estimate (KDE) of the predicted scores for the four diets. The bottom part shows the predicted scores (T_{pred}) from MCCV for each individual. **(B)** Boxplots of the four diets showing the predictions of the samples show a slight linear trend between the four diets using 2-h after lunch spot samples from negative to positive based on a Skillings-Mack test and subsequent Wilcoxon signed rank tests corrected by Hommel's method. **(C)** The data presented here is from the 5-h after lunch spot urine samples of day 3 predicted by the CS2 model, the key is the same as in panel (A). **(D)** Boxplots of the four diets showing the predictions of the samples show stronger linear trends between the four diets for the 5-h after lunch spot samples than for the 2-h after lunch samples. **(E)** The data presented here is from the 2-h after dinner spot urine samples of day 3 predicted by the CS3 model, the key is the same as in panel (A). **(F)** Boxplots of the four diets showing the predictions of the samples show linear trends between the four diets.

Supplementary References

1 Stamler J, Elliott P, Dennis B, et al. INTERMAP: background, aims, design, methods, and descriptive statistics (nondietary). J Hum Hypertens. 2003; **17**(9): 591-608.

2 Dennis B, Stamler J, Buzzard M, et al. INTERMAP: the dietary data - process and quality control. J Hum Hypertens. 2003; **17**(9): 609-22.

3 Fung TT, Chiuve SE, McCullough ML, Rexrode KM, Logroscino G, Hu FB. Adherence to a DASH-style diet and risk of coronary heart disease and stroke in women. Arch Intern Med. 2008; **168**(7): 713-20.

4 O'Brien MM, Kiely M, Galvin M, Flynn A. The importance of composite foods for estimates of vegetable and fruit intakes. Public health nutrition. 2003; **6**(7): 711-26.

5 Prynne CJ, Wagemakers JJ, Stephen AM, Wadsworth ME. Meat consumption after disaggregation of meat dishes in a cohort of British adults in 1989 and 1999 in relation to diet quality. Eur J Clin Nutr. 2009; **63**(5): 660-6.

6 Schofield WN. Predicting basal metabolic rate, new standards and review of previous work. Human nutrition Clinical nutrition. 1985; **39 Suppl 1**: 5-41.

7 Smith LM, Maher AD, Want EJ, et al. Large-Scale Human Metabolic Phenotyping and Molecular Epidemiological Studies-via H-1 NMR Spectroscopy of Urine: Investigation of Borate Preservation. Analytical chemistry. 2009; **81**(12): 4847-56.

8 Yang L, Ru GY, Tang HR, Liu CY. Human Urinary Metabolite Quantification in Nuclear Magnetic Resonance-based Metabonomics Using Electronic Internal Reference. Chinese J Anal Chem. 2010; **38**(6): 789-94.

9 Posma JM, Garcia-Perez I, De Iorio M, et al. Subset optimization by reference matching (STORM): an optimized statistical approach for recovery of metabolic biomarker structural information from 1H NMR spectra of biofluids. Analytical chemistry. 2012; **84**(24): 10694-701.

10 De Jong S. Simpls - an Alternative Approach to Partial Least-Squares Regression. Chemometr Intell Lab. 1993; **18**(3): 251-63.

11 Xu QS, Liang YZ. Monte Carlo cross validation. Chemometr Intell Lab. 2001; **56**(1): 1-11.

12 Efron B. Nonparametric Estimates of Standard Error - the Jackknife, the Bootstrap and Other Methods. Biometrika. 1981; **68**(3): 589-99.

13 Storey JD, Tibshirani R. Statistical significance for genomewide studies. Proc Natl Acad Sci U S A. 2003; **100**(16): 9440-5.

14 Hommel G. A Stagewise Rejective Multiple Test Procedure Based on a Modified Bonferroni Test. Biometrika. 1988; **75**(2): 383-6.

15 Stella C, Beckwith-Hall B, Cloarec O, et al. Susceptibility of human metabolic phenotypes to dietary modulation. J Proteome Res. 2006; **5**(10): 2780-8.

16 Svensson BG, Akesson B, Nilsson A, Paulsson K. Urinary-Excretion of Methylamines in Men with Varying Intake of Fish from the Baltic Sea. J Toxicol Env Health. 1994; **41**(4): 411-20.

17 Edmands WMB, Beckonert OP, Stella C, et al. Identification of Human Urinary Biomarkers of Cruciferous Vegetable Consumption by Metabonomic Profiling. J Proteome Res. 2011; **10**(10): 4513-21.

18 Heinzmann SS, Merrifield CA, Rezzi S, et al. Stability and Robustness of Human Metabolic Phenotypes in Response to Sequential Food Challenges. J Proteome Res. 2012; **11**(2): 643-55.

19 Ismail NA, Posma JM, Frost G, Holmes E, Garcia-Perez I. The role of metabonomics as a tool for augmenting nutritional information in epidemiological studies. Electrophoresis. 2013; **34**(19): 2776-86.

20 Lloyd AJ, Fave G, Beckmann M, et al. Use of mass spectrometry fingerprinting to identify urinary metabolites after consumption of specific foods. Am J Clin Nutr. 2011; **94**(4): 981-91.

21 Krupp D, Doberstein N, Shi L, Remer T. Hippuric Acid in 24-Hour Urine Collections Is a Potential Biomarker for Fruit and Vegetable Consumption in Healthy Children and Adolescents. The Journal of nutrition. 2012; **142**(7): 1314-20.

22 Garcia-Perez I, Posma JM, Chambers ES, et al. An Analytical Pipeline for Quantitative Characterization of Dietary Intake: Application To Assess Grape Intake. J Agric Food Chem. 2016; **64**(11): 2423-31.
23 Heinzmann SS, Brown IJ, Chan Q, et al. Metabolic profiling strategy for discovery of nutritional biomarkers: proline betaine as a marker of citrus consumption. Am J Clin Nutr. 2010; **92**(2): 436-43.
24 Lloyd AJ, Beckmann M, Fave G, Mathers JC, Draper J. Proline betaine and its biotransformation products in fasting urine samples are potential biomarkers of habitual citrus fruit consumption. The British journal of nutrition. 2011; **106**(6): 812-24.
25 World Health Organization. Global strategy on diet, physical activity and health. Geneva: WHO; 2004 (accessed 11/04/2016).

PROTOCOL

Dietary biomarker discovery using metabolomics

Main Sponsor	Imperial College London
Funders	Department of Investigative Medicine, Imperial College
REC Ref:	13/LO/0078

Protocol authorised by:

Professor Gary Frost,

Professor of Nutrition and Dietetics,

Department of Investigative Medicine,

Hammersmith Hospital Campus, Imperial College,

6th Floor, Commonwealth Building,

Du Cane Road, London,

W12 0NN

Dr. Edward Chambers

Research Associate

Department of Investigative Medicine,

Hammersmith Hospital Campus, Imperial College,

6th Floor, Commonwealth Building,

Du Cane Road, London,

W12 0NN

Study Management Group

Chief Investigator	Professor Gary Frost
Co-investigators	Dr. Edward Chambers
Statistician	Professor John Draper
Study Management	Professor Gary Frost

Sponsor

Imperial College is the main research sponsor for this study. For further information regarding the sponsorship conditions, please contact the Research Governance Manager at:

5th Floor,

Lab Block,

Charing Cross Hospital

Fulham Palace Road

London

W6 8RF

www.imperial.ac.uk/clinicalresearchoffice

PROBLEMS RELATED TO THIS TRIAL SHOULD BE REFERRED TO PROFESSOR GARY FROST

g.frost@imperial.ac.uk

Table of Contents

1. STUDY SUMMARY

2. INTRODUCTION

3. STUDY DESIGN

4. PARTICIPANT ENTRY

PRE-RANDOMISATION EVALUATIONS

INCLUSION CRITERIA

EXCLUSION CRITERIA

WITHDRAWAL CRITERIA

5. PHARMACOVIGILANCE

DEFINITIONS

REPORTING PROCEDURES

6. STATISTICS AND DATA ANALYSIS

7. REGULATORY ISSUES

8. PUBLICATION POLICY

1. STUDY SUMMARY

**TITLE**	Dietary biomarker discovery using metabolomics
**AIMS**	To identify chemicals in urine and blood associated with recent consumption of specific foods deemed to have high public health importance (oily fish, wholegrain foods, fruits and vegetables).
**DESIGN**	Participants will be asked to attend the NIHR/Wellcome Trust Imperial CRF at Hammersmith Hospital for 3-days, during 4 consecutive weeks. Each week, in a randomized order, participants will receive a diet with different amounts of specific test foods:<br>Week 1: 25% diet<br>Week 2: 50% diet<br>Week 3: 75% diet<br>Week 4: 100% diet
**POPULATION**	We will be studying healthy overweight men and women aged between 21 and 65 years.
**ELIGIBILITY**	Men and women with a body mass index of 20-35 aged between 21 to 65 years will be eligible to this study.
**DURATION**	4 weeks.

2. INTRODUCTION

Over the last 60 years, significant increases in longevity have been accompanied by a growing burden of age-related diseases including, coronary heart disease, type 2 diabetes and many cancers and a by dramatic increase in obesity prevalence. There is strong evidence that dietary choices modulate risk

of these diseases and strategies for reducing chronic disease burden emphasize the importance of changing dietary patterns (1, 2). Current public health guidelines have common themes that encourage consumption of specific food groups, for example oily fish, wholegrain foods, fruits and vegetables whilst reducing intakes of fatty and sugary foods.

A key factor in effective implementation of public health strategies is the need for validated population screening methods with which to determine the effectiveness of 'healthy eating' interventions in changing dietary habits. Further improvements in population health will require the development of evidence-based interventions to enhance consumption of specific foods and food groups to lower the risk of developing specific major chronic diseases and to promote lifelong health. These aspirations are predicated on the availability of robust tools for measuring dietary exposure.

Unfortunately the commonly used dietary exposure assessment methods (e.g. Food Frequency Questionnaires or diet diaries) are difficult to validate, subject to individual bias and depend upon food composition tables for estimation of intakes of energy, nutrients and other food constituents (3-5). Thus, it is difficult to assess if lack of effect of a healthy eating strategy is due to poor take up of the dietary advice, inadequate assessment methodology, or if the diet has no biological/health effect.

Significant advances have been made recently in developing biomarkers of dietary intake which are more objective and which are cost effective for larger studies and surveys. Use of urinary biomarkers is of particular interest as they reflect the end product of metabolism. However, chemical biomarkers are available for only a relatively small number of specific foods and food components and most are of uncertain validity (6-8).

Significant advances have been made recently in developing 'biomarkers' of dietary intake based on metabolites found in urine and blood but, currently, chemical biomarkers are available for only a relatively small number of specific foods and food components and most are of uncertain validity.

"Metabolomics" describes the measure of all (or many) of the metabolites (small molecules) in biological fluids such as blood or urine. Following digestion, absorption and metabolism, foods give rise to thousands of different metabolites in the human body and the appearance of certain metabolites is characteristic of particular foods.

REFERENCES

(1) Appel et al. (1997) N Engl J Med. 336, 1117-1124.

(2) Tuomilehto et al. (2001) N Engl J Med. 344, 1343-1350.

(3) Bingham et al. (1994) Br J Nutr 72, 619-643.

(4) Penn et al. (2010) Genes Nutr 5, 205-213.

(5) Milton (2007) Public Health Nutr 11, 1321-1335.

(6) Lee et al. (2006) Clin Chim Acta. 365, 264-269.

(7) Lovegrove et al. (2004) Am J Clin Nutr. 79, 974-982.

(8) Marklund et al. (2010) J Chromatogr B Analyt Technol Biomed Life Sci. 878, 888-894.

3. STUDY DESIGN

Aims:

1. To identify chemicals in urine and blood associated with recent consumption of specific foods deemed to have high public health importance (oily fish, wholegrain foods, fruits and vegetables).

2. To demonstrate a quantitative relationship between the amount of these specific foods eaten and each potential chemical 'biomarker' in urine and blood samples.

Methodology: Participants will undergo four 3-day interventions during 4 consecutive weeks, with the administration of specific diets differing in amounts of specific foods deemed to have high public health importance (oily fish, wholegrain foods, fruits and vegetables).

Participants: 30 healthy male and female volunteers, age 21-65 years, BMI 20-35 kg/m^2

Health Screening Visit

Participants will be clerked and examined by a research doctor. Measurements of blood pressure, an electrocardiogram (ECG) and blood samples (for full blood count, urea and electrolytes, liver function tests and lipid profile) will be taken. Height, weight, hip and waist measurements will be recorded. All women of child bearing age will have a pregnancy test.

Study visits

Participants will then undergo a 4 week study period. Participants will be asked to attend the NIHR/Wellcome Trust Imperial CRF at Hammersmith Hospital for 3-days, during 4 consecutive weeks. Participants will stay at the Clinical Investigations Unit for three nights and each week, in a randomized order, participants will receive a diet with different amounts of specific test foods deemed to have high public health importance (oily fish, wholegrain foods, fruits and vegetables):

Week 1: 25% diet

Week 2: 50% diet

Week 3: 75% diet

Week 4: 100% diet

Volunteers will arrive at approximately 8.30am on the first morning having fasted overnight. Volunteers will be given set menus for breakfast, lunch and evening meals. Urine (18 x 40 ml) and blood samples (6 x 10 ml) will be collected each 3-day study period and subjected to metabolomics analyses to investigate:

1. The quantitative relationship between the amount of test food consumed and biomarker abundance in urine and blood.
2. The kinetics of signal decay.

4. PARTICIPANT ENTRY

PRE-RANDOMISATION EVALUATIONS

Potential participants will first have a short telephone interview to assess their suitability for the study. Potential participants will then be interviewed and examined by one of the research doctors.

They will have blood tests and height, weight, hip and waist measurements. They will have an electrocardiogram (ECG). All women of child bearing age will have a pregnancy test.

INCLUSION CRITERIA

Healthy volunteers (body mass index (BMI) of 20-35 kg/m^2)

Age between 21-65 years (inclusive)

EXCLUSION CRITERIA

- Weight change of ≥ 3kg in the preceding 3 months
- Current smokers
- Substance abuse
- Excess alcohol intake
- Pregnancy
- Diabetes
- Cardiovascular disease
- Cancer
- Gastrointestinal disease e.g. inflammatory bowel disease or irritable bowel syndrome
- Kidney disease
- Liver disease
- Pancreatitis
- Use of medications likely to interfere with energy metabolism, appetite regulation and hormonal balance, including: anti inflammatory drugs or steroids, antibiotics, androgens, phenytoin, erythromycin or thyroid hormones.

Subjects with the above conditions would have an altered pattern of hormones and inflammatory molecules because of their disease process and would therefore give us confounding or misleading results.

WITHDRAWAL CRITERIA

The safety of the study participants takes priority. Any significant adverse event (as assessed by the researchers) will halt the study and the ethics committee and sponsor will be informed as per standard protocol. All adverse events will be recorded and investigators will review each adverse event as it

arises. In addition, participants will be free to withdraw at any time and are not required to give a reason.

5. PHARMACOVIGILANCE

Adverse Event (AE): Any untoward medical occurrence in a patient or clinical study subject.

Serious Adverse Event (SAE): Any untoward and unexpected medical occurrence that:

- results in death
- is life- threatening – refers to an event in which the subject was at risk of death at the time of the event; it does not refer to an event which hypothetically might have caused death if it was more severe.
- requires hospitalisation, or prolongation of existing inpatients' hospitalisation.
- results in persistent or significant disability or incapacity
- is a congenital abnormality or birth defect

Medical judgement should be exercised in deciding whether an AE is serious in other situations. Important AEs that are not immediately life threatening or do not result in death or hospitalisation but may jeopardise the subject or may require intervention to prevent one of the other outcomes listed in the definition above, should also be considered serious.

REPORTING PROCEDURES

All adverse events should be reported. Depending on the nature of the event the reporting procedures below should be followed. Any questions concerning adverse event reporting should be directed to the Chief Investigator in the first instance.

Non-serious AEs

All such events, whether expected or not, should be recorded.

Serious AEs (SEAs)

An SAE form should be completed and faxed to the Chief Investigator within 24 h. However, relapse, death and hospitalisations for elective treatment of a pre-existing condition do not need reporting as SAEs.

All SAEs should be reported to the xxxx Research Ethics Committee where in the opinion of the Chief Investigator the event was:

- 'related', i.e. resulted from the administration of any of the research procedures; and
- 'unexpected', i.e. an event that is not listed in the protocol as an expected occurrence.

Reports of related and unexpected SAEs should be submitted within 15 days of the Chief Investigator becoming aware of the event, using the NRES SAE form.

Local investigators should report any SAEs to the sponsor and their Local Research Ethics Committee and/ or Research and Development Office.

Contact details for reporting SAEs

Fax 020 838 33142, attention Professor Gary Frost

Please send SAE forms to Professor Gary Frost

6. STATISTICS AND DATA ANALYSIS

A formal power calculation is not possible as this will be the first study of its type. However, recent studies have demonstrated significant changes in metabolomics profiles in cohorts of 20 volunteers (1, 2). Allowing for a dropout rate of 33%, we intend to recruit 30 volunteers for this research.

An independent researcher (i.e. not linked to the study) will be given the task of randomisation, which will be by sealed envelopes.

REFERENCES

(1) Lloyd et al. (2011) Identification of urinary metabolites linked to the consumption of foods of high public health importance using mass spectrometry fingerprinting. American Journal of Clinical Nutrition. In Press, DOI: 10.3945/ajcn.111.017921.

(2) Lloyd et al. (2011) British J. Nutrition. 106, 812-824.

7. REGULATORY ISSUES

ETHICS APPROVAL

This study is awaiting ethical approval from the London Brent Research Ethics Committee. The study will be conducted in accordance with the recommendations for physicians involved in research on human subjects adopted by the 18th World Medical Assembly, Helsinki 1964 and later revisions. Amendments to the protocol should be approved by the sponsor before being sent to ethics. After ethical approval, all amendments must have Trust R&D approval before they can be implemented.

CONSENT

Consent to enter the study must be sought from each participant only after a full explanation has been given, an information leaflet offered, and time allowed for consideration. Signed participant consent should be obtained. The right of the participant to refuse to participate without giving reasons must be respected. After the participant has entered the study the clinician remains free to give alternative treatment to that specified in the protocol at any stage if he/she feels it is in the participant's best interest, but the reasons for doing so should be recorded. In such cases, the participants remain within

the study for the purposes of follow-up and data analyses. All participants are free to withdraw at any time from the study without giving reasons and without prejudicing further treatment.

CONFIDENTIALITY

The Chief Investigator will preserve the confidentiality of participants in the study and is registered under the Data Protection Act.

INDEMNITY

Imperial College holds negligent harm and non-negligent harm insurance policies, which apply to this study.

SPONSOR

Imperial College London will act as the main sponsor for this study. Delegated responsibilities will be assigned to the NHS trusts taking part in this study.

FUNDING

This research project is part of a grant funded by the Medical Research Council.

Participants will be reimbursed for their time. £600 will be awarded for completion of the entire study. Participants will be paid £150 for each of the 3-day interventions that they complete.

AUDITS AND INSPECTIONS

The study may be subject to inspection and audit by Imperial College London under their remit as sponsor and other regulatory bodies to ensure adherence to GCP and the NHS Research Governance Framework for Health and Social Care (2nd edition).

8. PUBLICATION POLICY

The findings of the research will be published in an open-access, peer-reviewed journal. In addition we will be collaborating with patient groups and professional groups to disseminate the findings via multiple media channels such as patient association publications, print and broadcast media.
